# Supplementary material for: Phylogenomics and the rise of the angiosperms
Source: Nature. 2024 Apr 24;629(8013):843–50. doi: 10.1038/s41586-024-07324-0 (PMC11111409; doi:10.1038/s41586-024-07324-0)
Supplement: Supplementary file 1 — Supplementary Methods, Results, Discussion, references, Figs. 1–24, captions for Tables 1–8, Files 1 and 2 and extended acknowledgements. [file 41586_2024_7324_MOESM1_ESM.pdf]

nature portfolio

https://doi.org/10.1038/s41586-024-07324-0

Supplementary information

Phylogenomics and the rise of the angiosperms

In the format provided by the authors and unedited

Phylogenomics and the rise of the angiosperms

Alexandre R. Zuntini[1,138], Tom Carruthers[1,138], Olivier Maurin[1], Paul C. Bailey[1], Kevin Leempoel[1], Grace E. Brewer[1], Niroshini Epitawalage[1], Elaine Françoso[1,2], Berta Gallego-Paramo[1], Catherine McGinnie[1], Raquel Negrão[1], Shyamali R. Roy[1], Lalita Simpson[3], Eduardo Toledo Romero[1], Vanessa M. A. Barber[1], Laura Botigué[4], James J. Clarkson[1], Robyn S. Cowan[1], Steven Dodsworth[5], Matthew G. Johnson[6], Jan T. Kim[7], Lisa Pokorny[1,8], Norman J. Wickett[9], Guilherme M. Antar[10,11], Lucinda DeBolt[12], Karime Gutierrez[12], Kasper P. Hendriks[13,14], Alina Hoewener[15], Ai-Qun Hu[1], Elizabeth M. Joyce[3,16], Izai A. B. S. Kikuchi[17], Isabel Larridon[1], Drew A. Larson[18], Elton John de Lírio[10], Jing-Xia Liu[19], Panagiota Malakasi[1], Natalia A. S. Przelomska[1,5], Toral Shah[1], Juan Viruel[1], Theodore R. Allnutt[20], Gabriel K. Ameka[21], Rose L. Andrew[22], Marc S. Appelhans[23], Montserrat Arista[24], María Jesús Ariza[25], Juan Arroyo[24], Watchara Arthan[1], Julien B. Bachelier[26], C. Donovan Bailey[27], Helen F. Barnes[20], Matthew D. Barrett[3], Russell L. Barrett[28], Randall J. Bayer[29], Michael J. Bayly[30], Ed Biffin[31], Nicky Biggs[1], Joanne L. Birch[30], Diego Bogarín[14,32], Renata Borosova[1], Alexander M. C. Bowles[33], Peter C. Boyce[34], Gemma L. C. Bramley[1], Marie Briggs[1], Linda Broadhurst[35], Gillian K. Brown[36], Jeremy J. Bruhl[22], Anne Bruneau[37], Sven Buerki[38], Edie Burns[1], Margaret Byrne[39], Stuart Cable[1], Ainsley Calladine[31], Martin W. Callmander[40], Ángela Cano[41], David J Cantrill[20], Warren M. Cardinal-McTeague[42], Mónica M. Carlsen[43], Abigail J. A. Carruthers[1], Alejandra de Castro Mateo[24], Mark W. Chase[1,44], Lars W. Chatrou[45], Martin Cheek[1], Shilin Chen[46,47], Maarten J. M. Christenhusz[1,48,49], Pascal-Antoine Christin[50], Mark A. Clements[35], Skye C. Coffey[51], John G. Conran[52], Xavier Cornejo[53], Thomas L. P. Couvreur[54], Ian D. Cowie[55], Laszlo Csiba[1], Iain Darbyshire[1], Gerrit Davidse[43], Nina M. J. Davies[1], Aaron P. Davis[1], Kor-jent van Dijk[56], Stephen R. Downie[57], Marco F. Duretto[28], Melvin R. Duvall[58], Sara L. Edwards[1], Urs Eggli[59], Roy H. J. Erkens[14,60,61], Marcial Escudero[24], Manuel de la Estrella[62], Federico Fabriani[45], Michael F. Fay[1], Paola de L. Ferreira[63,64], Sarah Z. Ficinski[1], Rachael M. Fowler[30], Sue Frisby[1], Lin Fu[65], Tim Fulcher[1], Mercè Galbany-Casals[66], Elliot M. Gardner[67], Dmitry A. German[68], Augusto Giaretta[69],

Marc Gibernau[70], Lynn J. Gillespie[71], Cynthia C. González[72], David J. Goyder[1], Sean W. Graham[17], Aurélie Grall[1], Laura Green[1], Bee F. Gunn[20], Diego G. Gutiérrez[73], Jan Hackel[1,74], Thomas Haevermans[75], Anna Haigh[1], Jocelyn C. Hall[76], Tony Hall[1], Melissa J. Harrison[3], Sebastian A. Hatt[1], Oriane Hidalgo[77], Trevor R. Hodkinson[78], Gareth D. Holmes[20], Helen C. F. Hopkins[1], Christopher J. Jackson[20], Shelley A. James[51], Richard W. Jobson[28], Gudrun Kadereit[79], Imalka M. Kahandawala[1], Kent Kainulainen[80], Masahiro Kato[81], Elizabeth A. Kellogg[82], Graham J. King[83], Beata Klejevskaja[84], Bente B. Klitgaard[1], Ronell R. Klopper[85,86], Sandra Knapp[87], Marcus A. Koch[88], James H. Leebens-Mack[89], Frederic Lens[14], Christine J. Leon[1], Étienne Léveillé-Bourret[90], Gwilym P. Lewis[1], De-Zhu Li[19], Lan Li[91], Sigrid Liede-Schumann[92], Tatyana Livshultz[93,94], David Lorence[95], Meng Lu[1], Patricia Lu-Irving[28], Jaquelini Luber[96], Eve J. Lucas[1], Manuel Luján[1], Mabel Lum[97], Terry D. Macfarlane[51], Carlos Magdalena[1], Vidal F. Mansano[96], Lizo E. Masters[1], Simon J. Mayo[1], Kristina McColl[28], Angela J. McDonnell[98], Andrew E. McDougall[56], Todd G. B. McLay[20], Hannah McPherson[28], Rosa I. Meneses[99], Vincent S. F. T. Merckx[14], Fabián A. Michelangeli[100], John D Mitchell[100], Alexandre K. Monro[1], Michael J. Moore[101], Taryn L. Mueller[102], Klaus Mummenhoff[13], Jérôme Munzinger[103], Priscilla Muriel[104], Daniel J. Murphy[20], Katharina Nargar[3,35], Lars Nauheimer[3], Francis J. Nge[31], Reto Nyffeler[105], Andrés Orejuela[106,107], Edgardo M. Ortiz[15], Luis Palazzesi[73], Ariane Luna Peixoto[96], Susan K. Pell[108], Jaume Pellicer[77], Darin S. Penneys[109], Oscar A. Perez-Escobar[1], Claes Persson[110], Marc Pignal[75], Yohan Pillon[111], José R. Pirani[10], Gregory M. Plunkett[100], Robyn F. Powell[1], Ghillean T. Prance[1], Carmen Puglisi[1,43], Ming Qin[65], Richard K. Rabeler[18], Paul E. J. Rees[1], Matthew Renner[28], Eric H. Roalson[112], Michele Rodda[113], Zachary S. Rogers[114], Saba Rokni[1], Rolf Rutishauser[105], Miguel F. de Salas[115], Hanno Schaefer[15], Rowan J. Schley[116], Alexander Schmidt-Lebuhn[35], Alison Shapcott[117], Ihsan Al-Shehbaz[43], Kelly A. Shepherd[51], Mark P. Simmons[118], Andre O. Simões[119], Ana Rita G. Simões[1], Michelle Siros[1,120], Eric C. Smidt[121], James F. Smith[38], Neil Snow[122], Douglas E. Soltis[123], Pamela S. Soltis[123], Robert J. Soreng[124], Cynthia A. Sothers[1], Julian R. Starr[125], Peter F. Stevens[43], Shannon C. K. Straub[126], Lena Struwe[127], Jennifer M. Taylor[91], Ian R. H. Telford[22], Andrew H. Thornhill[22,31,52], Ifeanna Tooth[28], Anna Trias-Blasi[1], Frank Udovicic[20], Timothy M. A. Utteridge[1], Jose C. Del Valle[24], G. Anthony Verboom[128], Helen P. Vonow[31], Maria S. Vorontsova[1], Jurriaan M. de Vos[129], Noor

Al-Wattar[1], Michelle Waycott[31,52], Cassiano A. D. Welker[130], Adam J. White[131], Jan J. Wieringa[14], Luis T. Williamson[56], Trevor C. Wilson[28], Sin Yeng Wong[132], Lisa A. Woods[28], Roseina Woods[1], Stuart Worboys[3], Martin Xanthos[1], Ya Yang[133], Yu-Xiao Zhang[134], Meng-Yuan Zhou[19], Sue Zmarzty[1], Fernando O. Zuloaga[135], Alexandre Antonelli[1,110,136,137], Sidonie Bellot[1], Darren M. Crayn[3], Olwen M. Grace[1,106], Paul J. Kersey[1], Ilia J. Leitch[1], Hervé Sauquet[28], Stephen A. Smith[18,139], Wolf L. Eiserhardt[1,64,139], Félix Forest[1,139] and William J. Baker[1,139,*]

[1]Royal Botanic Gardens, Kew, Richmond, UK
[2]Centre for Ecology, Evolution and Behaviour, Department of Biological Sciences, School of Life Sciences and the Environment, Royal Holloway University of London, Egham, UK
[3]Australian Tropical Herbarium, James Cook University, Smithfield, Queensland, Australia
[4]Centre for Research in Agricultural Genomics (CRAG), CSIC-IRTA-UAB-UB, Campus UAB, Bellaterra, Barcelona, Spain
[5]School of Biological Sciences, University of Portsmouth, Portsmouth, UK
[6]Texas Tech University, Lubbock, TX, USA
[7]School of Physics, Engineering and Computer Science, University of Hertfordshire, Hatfield, UK
[8]Department of Biodiversity and Conservation, Real Jardín Botánico (RJB-CSIC), Madrid, Spain
[9]Department of Biological Sciences, Clemson University, Clemson, SC, USA
[10]Departamento de Botânica, Instituto de Biociências, Universidade de São Paulo, São Paulo, São Paulo, Brazil
[11]Departamento de Ciências Agrárias e Biológicas, Centro Universitário Norte do Espírito Santo, Universidade Federal do Espírito Santo, São Mateus, Espírito Santo, Brazil
[12]Smith College, Northampton, MA, USA
[13]Department of Biology, University of Osnabrück, Osnabrück, Germany
[14]Naturalis Biodiversity Center, Leiden, Netherlands
[15]Plant Biodiversity, Technical University Munich, Freising, Bayern, Germany

[16]Systematic, Biodiversity and Evolution of Plants, Ludwig Maximilian University of Munich, Munich, Bayern, Germany
[17]Department of Botany, University of British Columbia, Vancouver, British Columbia, Canada
[18]Department of Ecology & Evolutionary Biology, University of Michigan, Ann Arbor, MI, USA
[19]Germplasm Bank of Wild Species, Kunming Institute of Botany, Chinese Academy of Sciences, Kunming, Yunnan, China
[20]Royal Botanic Gardens Victoria, Melbourne, Victoria, Australia
[21]Department of Plant and Environmental Biology, University of Ghana, Legon, Accra, Ghana
[22]Botany and N.C.W. Beadle Herbarium, University of New England, New South Wales, Australia
[23]Department of Systematics, Biodiversity and Evolution of Plants, Albrecht-von-Haller Institute of Plant Sciences, University of Göttingen, Göttingen, Germany
[24]Departamento de Biología Vegetal y Ecología, Facultad de Biología, Universidad de Sevilla, Seville, Spain
[25]General Research Services, Herbario SEV, CITIUS, Universidad de Sevilla, Seville, Spain
[26]Institute of Biology, Freie Universität, Berlin, Germany
[27]Department of Biology, New Mexico State University, Las Cruces, NM, USA
[28]National Herbarium of NSW, Botanic Gardens of Sydney, Mount Annan, New South Wales, Australia
[29]Department of Biological Sciences, University of Memphis, Memphis, TN, USA
[30]School of BioSciences, The University of Melbourne, Parkville, Victoria, Australia
[31]State Herbarium of South Australia, Botanic Gardens and State Herbarium, Adelaide, South Australia, Australia
[32]Jardín Botánico Lankester, Universidad de Costa Rica,Cartago, Costa Rica
[33]School of Geographical Sciences, University of Bristol, Bristol, UK
[34]Centro Studi Erbario Tropicale, Dipartimento di Biologia, University of Florence, Firenze, Italy
[35]Centre for Australian National Biodiversity Research, National Research Collections Australia, CSIRO, Canberra, Australian Capital Territory, Australia

[36]Queensland Herbarium, Department of Environment and Science, Toowong, Queensland, Australia
[37]Institut de recherche en biologie végétale and Département de Sciences biologiques, University of Montreal, Montreal, Quebec, Canada
[38]Department of Biological Sciences, Boise State University, Boise, ID, USA
[39]Biodiversity and Conservation Science, Department of Biodiversity, Conservation and Attractions, Kensington, Western Australia, Australia
[40]Conservatoire et Jardin botaniques de Genève, Chambésy, Switzerland
[41]Cambridge University Botanic Garden, Cambridge, UK
[42]Department of Forest and Conservation Sciences, University of British Columbia, Vancouver, British Columbia, Canada
[43]Missouri Botanical Garden, St. Louis, MO, USA
[44]Department of Environment and Agriculture, Curtin University, Bentley, Western Australia, Australia
[45]Department of Biology, Ghent University, Ghent, Belgium
[46]Institute of Herbgenomics, Chengdu University of Traditional Chinese medicine, Chengdu, Sichuan, China
[47]Institute of medicinal plant development, Chinese academy of medical sciences, Beijing, China
[48]Department of Environment and Agriculture, Curtin University, Perth, Western Australia, Australia
[49] Plant Gateway, Den Haag, Netherlands
[50]Ecology and Evolutionary Biology, School of Biosciences, University of Sheffield, Sheffield, UK
[51]Western Australian Herbarium, Department of Biodiversity, Conservation and Attractions, Kensington, Western Australia, Australia
[52]School of Biological Sciences, The University of Adelaide, Adelaide, South Australia, Australia
[53]Herbario GUAY, Facultad de Ciencias Naturales, Universidad de Guayaquil, Guayaquil, Ecuador
[54]DIADE, Université Montpellier, CIRAD, IRD, Montpellier, France
[55]Northern Territory Herbarium, Department of Environment, Parks & Water Security, Palmerston, Northern Territory, Australia

[56]The University of Adelaide, North Terrace, South Australia, Australia
[57]Department of Plant Biology, University of Illinois at Urbana-Champaign, Urbana, IL, USA
[58]Department of Biological Sciences and Institute for the Study of the Environment, Sustainability, and Energy, Northern Illinois University, DeKalb, IL, USA
[59]Sukkulenten-Sammlung Zürich / Grün Stadt Zürich, Zürich, Switzerland
[60]Maastricht Science Programme, Maastricht University, Maastricht, Netherlands
[61]System Earth Science, Maastricht University, Venlo, Netherlands
[62]Departamento de Botánica, Ecología y Fisiología Vegetal, Facultad de Ciencias, Universidad de Córdoba, Córdoba, Spain
[63]Departamento de Biologia, Faculdade de Ciências e Letras de Ribeirão Preto, Universidade de São Paulo, São Paulo, São Paulo, Brazil
[64]Department of Biology, Aarhus University, Aarhus C, Denmark
[65]South China Botanical Garden, Chinese Academy of Sciences, Guangzhou, Guangdong, China
[66]Systematics and Evolution of Vascular Plants (UAB) – Associated Unit to CSIC by IBB, Departament de Biologia Animal, Biologia Vegetal i Ecologia, Facultat de Biociències, Universitat Autònoma de Barcelona, Bellaterra, Spain
[67]Department of Biology, Case Western Reserve University, Cleveland, OH, USA
[68]Altai State University, Barnaul, Russia
[69]Faculdade de Ciências Biológicas e Ambientais, Universidade Federal da Grande Dourados, Dourados, Mato Grosso do Sul, Brazil
[70]Laboratoire Sciences Pour l'Environnement, Université de Corse, Ajaccio, Corsica, France
[71]Canadian Museum of Nature, Ottawa, Ontario, Canada
[72]Herbario Trelew, Universidad Nacional de la Patagonia San Juan Bosco, Trelew, Chubut, Argentina
[73]Museo Argentino de Ciencias Naturales (MACN-CONICET), Buenos Aires, Argentina
[74]Department of Biology, Universität Marburg, Marburg, Germany
[75]Institut de Systématique, Evolution, Biodiversité, Muséum National d'Histoire Naturelle, Paris, France
[76]Department of Biological Sciences, University of Alberta, Edmonton, Alberta, Canada
[77]Institut Botànic de Barcelona (IBB, CSIC-Ajuntament de Barcelona), Barcelona, Spain

[78]Botany, School of Natural Sciences, Trinity College Dublin, The University of Dublin, Dublin, Ireland
[79]Prinzessin Therese von Bayern-Lehrstuhl für Systematik, Biodiversität & Evolution der Pflanzen, Ludwig-Maximilians-Universität München, Botanische Staatssammlung München, Botanischer Garten München-Nymphenburg, Munich, Bayern, Germany
[80]Gothenburg Botanical Garden, Gothenburg, Sweden
[81]National Museum of Nature and Science, Tsukuba, Japan
[82]Donald Danforth Plant Science Center, St. Louis, MO, USA
[83]Southern Cross University, Lismore, New South Wales, Australia
[84]Synergy SRG, Luton, Bedfordshire, UK
[85]Foundational Biodiversity Science Division, South African National Biodiversity Institute, Pretoria, South Africa
[86]Department of Plant and Soil Sciences, University of Pretoria, Pretoria, South Africa
[87]Natural History Museum, London, UK
[88]Centre for Organismal Studies, Biodiversity and Plant Systematics, Heidelberg University, Heidelberg, Germany
[89]Department of Plant Biology, University of Georgia, Athens, GA, USA
[90]Institut de Recherche en Biologie Végétale, University of Montreal, Montreal, Quebec, Canada
[91]CSIRO, Canberra, Australian Capital Territory, Australia
[92]Department of Plant Systematics, University of Bayreuth, Bayreuth, Germany
[93]Department of Biodiversity, Earth, and Environmental Sciences, Drexel University, Philadelphia, PA, USA
[94]Academy of Natural Science, Drexel University, Philadelphia, PA, USA
[95]National Tropical Botanical Garden, Kalaheo, HI, USA
[96]Instituto de Pesquisas Jardim Botânico do Rio de Janeiro, Rio de Janeiro, Rio de Janeiro, Brazil
[97]Bioplatforms Australia Ltd., Sydney, New South Wales, Australia
[98]Department of Biological Sciences, Saint Cloud State University, Saint Cloud, MN, USA
[99]Instituto de Arqueología y Antropología, Universidad Católica del Norte, San Pedro de Atacama, Chile
[100]New York Botanical Garden, Bronx, NY, USA

[101]Department of Biology, Oberlin College, Oberlin, OH, USA
[102]Department of Ecology, Evolution, & Behavior, University of Minnesota, St. Paul, MN, USA
[103]AMAP, Université Montpellier, IRD, CIRAD, CNRS, INRAE, Montpellier, France
[104]Laboratorio de Ecofisiología, Escuela de Ciencias Biológicas, Pontificia Universidad Católica del Ecuador, Quito, Ecuador
[105]Department of Systematic and Evolutionary Botany, University of Zürich, Zürich, Switzerland
[106]Royal Botanic Garden Edinburgh, Edinburgh, UK
[107]Grupo de Investigación en Recursos Naturales Amazónicos, Instituto Tecnológico del Putumayo, Mocoa, Putumayo, Colombia
[108]U.S. Botanic Garden, Washington DC, USA
[109]Department of Biology and Marine Biology, University of North Carolina Wilmington, Wilmington, NC, USA
[110]Department of Biological and Environmental Sciences, University of Gothenburg, Gothenburg, Sweden
[111]LSTM, Université Montpellier, CIRAD, IRD, Montpellier, France
[112]School of Biological Sciences, Washington State University, Pullman, WA, USA
[113]National Parks Board, Singapore Botanic Gardens, Singapore
[114]New Mexico State University, Las Cruces, NM, USA
[115]Tasmanian Herbarium, University of Tasmania, Sandy Bay, Tasmania, Australia
[116]University of Exeter, Exeter, UK
[117]School of Science Technology and Engineering, Center for Bioinnovation, University Sunshine Coast, Queensland, Australia
[118]Department of Biology, Colorado State University, Fort Collins, CO, USA
[119]Departamento de Biologia Vegetal, Universidade Estadual de Campinas, Campinas, São Paulo, Brazil
[120]University of California - San Francisco, San Francisco, CA, USA
[121]Departamento de Botânica, Universidade Federal do Paraná, Curitiba, Paraná, Brazil
[122]Pittsburg State University, Pittsburg, KS, USA
[123]Florida Museum of Natural History, University of Florida, Gainesville, FL, USA
[124]Smithsonian Institution, Washington DC, USA
[125]Department of Biology, University of Ottawa, Ottawa, Ontario, Canada

[126]Hobart and William Smith Colleges, Geneva, NY, USA
[127]Rutgers University, New Brunswick, NJ, USA
[128]Department of Biological Sciences and Bolus Herbarium, University of Cape Town, Cape Town, South Africa
[129]Department of Environmental Sciences - Botany, University of Basel, Basel, Switzerland
[130]Instituto de Biologia, Universidade Federal de Uberlândia, Uberlândia, Minas Gerais, Brazil
[131]Australian National Herbarium, Centre for Australian National Biodiversity Research, National Research Collections Australia, CSIRO, Canberra, Australian Capital Territory, Australia
[132]Institute of Biodiversity And Environmental Conservation, Universiti Malaysia Sarawak, Samarahan, Sarawak, Malaysia
[133]University of Minnesota-Twin Cities, St. Paul, MN, USA
[134]Southwest Forestry University, Kunming, Yunnan, China
[135]Instituto de Botánica Darwinion, San Isidro, Buenos Aires, Argentina
[136]Gothenburg Global Biodiversity Centre, University of Gothenburg, Gothenburg, Sweden
[137]Department of Biology, University of Oxford, Oxford, UK
[138]These authors contributed equally: Alexandre R. Zuntini, Tom Carruthers
[139]These authors jointly supervised this work: Stephen A. Smith, Wolf L. Eiserhardt, Felix Forest and William J. Baker
[*]e-mail: w.baker@kew.org

Index

Supplementary methods..12
Divergence time estimation.. 12
Molecular rate variation and conflict.. 12
Fossil calibrations... 14
Diversification rate estimation...14
Accounting for divergence time estimation uncertainty..14
Time-dependent diversification rate estimation.. 15
Lineage-specific diversification rate estimation.. 17
Supplementary results and discussion... 17
Time-dependent diversification rate estimation... 18
Clarifying the relationship between diversification and gene tree conflict.............................19
Additional references...20
Supplementary figures...23
Supplementary Fig. 1 | Workflow diagram of phylogenetic inference pipeline......................24
Supplementary Fig. 2 | Backbone species tree... 25
Supplementary Fig. 3 | Global species tree..26
Supplementary Fig. 4 | Tanglegram comparing relationships among angiosperm families in this study with Li et al. (2021)...27
Supplementary Fig. 5 | Lineage through time plots for the eight time-calibrated phylogenetic trees 28
Supplementary Fig. 6 | Percentage of extant lineages sampled through time 29
Supplementary Fig. 7 | Simulations exploring the relationship between gene tree conflict and diversification 30
Supplementary Fig. 8 | Gene tree conflict across the phylogeny.. 31
Supplementary Fig. 9 | Heatmap of relative gene recovery per order................................. 32
Supplementary Fig. 10 | Pairwise comparison of normalised Robinson-Foulds distances between the backbone tree and replicates...33
Supplementary Fig. 11 | Heatmap of gene occupancy in alignments per order................... 34
Supplementary Fig. 12 | Phylogenetic distribution of fossil calibrations............................... 35
Supplementary Fig. 13 | Comparison of speciation rate, extinction rate, and net diversification rate estimates from different time dependent diversification rate models that were used in this study... 36
Supplementary Fig. 14 | Summary of lineage-specific diversification rate shifts estimated by BAMM for the young tree..37
Supplementary Fig. 15 | Summary of lineage-specific diversification rate shifts estimated by RevBayes for the young tree... 38
Supplementary Fig. 16 | Summary of lineage-specific diversification rate shifts estimated by RevBayes for the young tree... 39
Supplementary Fig. 17 | Summary of lineage-specific diversification rate shifts estimated by

RevBayes for the old tree........ 40
Supplementary Fig. 18 | Net diversification rates estimated in BAMM for the young tree..... 41
Supplementary Fig. 19 | Net diversification rates estimated in BAMM for the old tree.......... 42
Supplementary Fig. 20 | Net diversification rates estimated in BAMM for the young tree..... 43
Supplementary Fig. 21 | Net diversification rates estimated in RevBayes for the young tree........ 44
Supplementary Fig. 22 | Net diversification rates estimated in RevBayes for the old tree.... 45
Supplementary Fig. 23 | Net diversification rates estimated in RevBayes for the young tree........ 46
Supplementary Fig. 24 | The number of large speciation rate increases through time in the young tree........ 47
Supplementary tables........ 48
Supplementary Tab. 1 | Sample metadata and recovery statistics........ 48
Supplementary Tab. 2 | Details of plant portraits illustrating Fig. 1........ 48
Supplementary Tab. 3 | Ages of major clades, orders and families........ 48
Supplementary Tab. 4 | Number of rate shifts per order........ 49
Supplementary Tab. 5 | Gene alignments summary and SortaDate results........ 49
Supplementary Tab. 6 | Fossil calibration dataset – AngioCal v1.1........ 49
Supplementary Tab. 7 | Fossil calibrations points used in this study........ 49
Supplementary Tab. 8 | Bayes factor comparison for different time variable diversification rate models........ 49
Supplementary files........ 50
Supplementary File 1 | Target file used for sequence recovery........ 50
Supplementary File 2 | Fossil calibration dataset – AngioCal v1.1........ 50
Extended acknowledgements........ 51

Supplementary methods

Divergence time and diversification rate estimates were essential for the interpretation of our phylogeny in a macroevolutionary context. However, both fields have been at the centre of recent methodological debate[e.g.,82,85] and appear to be especially prone to misleading inferences if methodological limitations are not adequately accounted for. Below, we provide further detail on the methodological steps that we took in our analyses, and describe how these steps helped us to account for the limitations that exist in current methods.

Divergence time estimation

Divergence time estimates are sensitive to the molecular clock model[86,87], the extent of topological incongruence between gene trees and the species tree[79,88], and fossil calibrations[5,89]. Here, we provide further detail on how we accounted for these issues in the divergence time analyses we performed in treePL[76].

Molecular rate variation and conflict

The approach we selected (penalised likelihood implemented in treePL) requires an input tree with branch lengths equal to the number of substitutions per site (molecular branch lengths) and, using an autocorrelated molecular clock model, estimates substitution rates and divergence times based on this input. Estimation of molecular branch lengths typically involves the analysis of a concatenated alignment of multiple loci. Two necessary considerations when analysing multiple loci are 1) whether gene trees for individual loci are topologically incongruent with the species tree; and 2) whether some loci exhibit stronger variation in substitution rate among branches. Both of these issues can cause erroneous divergence time estimates: topological incongruence can jeopardise molecular branch length estimates[79,88], and strong patterns of among-branch substitution rate variation undermine the implementation of molecular clock models. This latter problem is especially important if there are consistent rate differences between branches across the analysed loci, because it causes

lineage-specific rates, a source of error in divergence time estimation regardless of the quantity of sampled data[90].

We used several approaches to explore these issues in our dataset. First, SortaDate[77] was used to rank loci, prioritising the number of congruent bipartitions with the species tree topology (Supplementary Tab. 5). Molecular branch lengths were then estimated in the species tree with the top 25 and 50 loci selected by SortaDate. Inclusion of 50 loci caused a small inflation of molecular branch length estimates on terminal branches by around 1.8% relative to 25 loci (an expected pattern when loci with topologically incongruent gene trees are analysed)[79,88]. Furthermore, using 50 loci led to the incorporation of just one additional tip in the species tree (*Gevuina avellana*, Proteaceae). Given the marginal benefits of including 50 loci with respect to species sampling, alongside a notable (although small) inflation of branch length estimates for terminal branches, subsequent divergence time estimates were based on 25 loci.

Branch lengths in the species tree was also estimated according to the "congruent branches" method of Carruthers et al.[79]. In this method, for each branch in the species tree, branch length estimates are based on the mean branch length across all gene trees with a congruent bipartition for the species tree branch. Topologically incongruent parts of gene trees are therefore excluded from branch length estimation in the species tree. This method produced similar molecular branch length estimates to those based on analysis of a concatenated alignment of the top 25 loci from SortaDate. Subsequently, molecular branch length estimates based on the top 25 loci from SortaDate were used in divergence time analyses.

Our implementation of SortaDate and the "congruent branches" method primarily refer to different ways of accounting for topological incongruence between gene trees and the species tree. Even though SortaDate measures root-to-tip variation (a proxy for substitution rate variation), our ranking of gene trees prioritised topological congruence with the species tree, rather than "clock-like" loci. Therefore, to understand the potential effects of among-branch-substitution-rate variation that inevitably exist in our dataset, we experimented with different smoothing values in treePL (0.1, 1, 10, and 100). We did

not however perform cross-validation to identify the optimum smoothing value, because this was not computationally feasible with the size of the dataset.

Fossil calibrations

Fossil calibrations were based on the AngioCal dataset of fossil calibrations originally assembled by Ramírez-Barahona et al.[5]. The phylogenetic placement of all fossils within this dataset was re-evaluated in the context of the species tree estimated here. This re-evaluation referred explicitly to the placement of fossils within the estimated species tree, not the identity of the fossil. Alongside this, minor updates to the underlying fossil dataset were made, with these changes being presented in an updated version of the dataset – AngioCal.v1.1 (Supplementary Tab. 6, Supplementary File 2). The final set of fossil calibrations that were used is available (Supplementary Tab. 7), with the phylogenetic placement of each fossil being displayed in Supplementary Fig. 12.

Diversification rate estimation

Diversification rate estimates are dependent on accurate divergence time estimates. Alongside this, further theoretical issues relate specifically to diversification rate estimation. This includes problems of model identifiability in time-dependent diversification rate models[82,91,92], and prior sensitivity and accounting for diversification rate shifts on extinct lineages in lineage specific models[92-94]. Below, we outline how we accounted for these issues.

Accounting for divergence time estimation uncertainty

Varying the maximum constraint at the angiosperm crown node between either 154 Ma or 247 Ma led to substantially different divergence time estimates throughout angiosperms (Extended Fig. 2; Supplementary Fig. 5). Nodes at all depths were considerably older with a maximum constraint of 247 Ma. By contrast, varying the smoothing value used in treePL tended to have a more limited effect (Extended Fig. 2; Supplementary Fig. 5). In this case, significant age differences were restricted to

younger nodes, there was no directional bias in these age differences, and the overall pattern of lineage accumulation through time (a proxy for the temporal distribution of divergence times throughout the tree) remained the same (Supplementary Fig. 5). The similarity of age estimates with different smoothing values highlights that not performing cross-validation analyses to identify the optimum smoothing value is likely to be relatively unimportant for this study.

Given that substantially different divergence time estimates are likely to affect diversification rate estimates, we estimated diversification rates on trees estimated with both maximum constraints, but only used trees estimated with a smoothing value of 10. This smoothing value leads to the estimation of moderate levels of variation in substitution rates among branches (four-fold rate differences were incorporated by two standard deviations around the mean estimated rate). Importantly, these two different maximum constraints do not affect the overall pattern of diversification rate shifts in our tree, although there is some impact on the absolute timing of when shifts occur (Figs. 3-4; Extended Figs. 5-6).

Time-dependent diversification rate estimation

We estimated time-dependent rates of speciation, extinction, and net diversification to enable insights into broad scale patterns of angiosperm diversification through time. Limitations of time-dependent diversification analyses have been known for decades[95], and have recently received further attention[82,91,92]. These limitations exist because a myriad of time-varying speciation and extinction rates can have an equal likelihood for a given time-calibrated phylogeny. This presents a challenge for estimating and interpreting time-dependent patterns of diversification. Alongside this, the implementation of time-dependent models at deep positions in a phylogeny can be problematic, because the amount of data (branches) over which rates are estimated decreases exponentially into the past[91].

To prevent these issues from undermining our analyses and the conclusions we drew from them, we took the following general approach. First, we explicitly delimited the purpose of each analysis. This provided a basis for evaluating whether the methods

we employed, despite their inevitable imperfections, were sufficient for their purpose. Second, we compared models to evaluate which parameters were identifiable and which models were likely to be equally congruent with the time-calibrated phylogenies. Finally, we interpreted time-dependent diversification rate estimates in their broader biological context, such as their relationship with gene tree conflict, the temporal distribution of lineage specific speciation rate shifts, or the origination of major taxonomic groups such as orders or families. This enabled an assessment of confidence in our inferences and the conclusions that we drew from them.

Initial time-dependent diversification rate analyses were primarily exploratory. Given that no previous study has analysed angiosperm diversification rates in a dataset of this scale, we wanted to determine whether there was evidence of significant trends in diversification dynamics over geological timescales. We therefore estimated diversification rate parameters using three different models: 1) a "null" model with constant rates of speciation and extinction through time, 2) a model that allowed speciation rates to vary between time intervals but had a constant rate of extinction, and 3) a model that allowed extinction rates to vary between time intervals but had a constant rate of speciation. The latter two models enabled exploration of how time-varying patterns of either speciation or extinction explain the accumulation of angiosperm diversity through time. In the time-varying analyses, parameters were estimated within 5 Ma intervals, but these intervals were extended where necessary to ensure that there were at least 50 branching events within each time interval. This meant there were sufficient data points within each interval for rate estimation.

The time-variable models estimate significant differences (non-overlapping 95% highest posterior densities) in net diversification rate between time intervals, including an early burst and a surge during the Cenozoic (Fig. 3; Extended Fig. 5; Supplementary Fig. 13). We sought to clarify the support for this general pattern and determine whether it was possible to distinguish between models that explain it by changes to the speciation rate or changes to the extinction rate. We therefore performed Bayes factor comparison between models in RevBayes using a stepping stone sampler. We compared three different models: 1) constant rates of speciation and extinction through

time, 2) variable speciation rates but constant extinction, and 3) variable extinction rates but constant speciation (for details of models compared see Supplementary Tab. 8).

Lineage-specific diversification rate estimation

Our main lineage specific diversification rate analyses were performed in BAMM[83]. This method has been criticised for the following reasons in Moore et al.[93]: 1) diversification rate shifts on extinct lineages are not adequately accounted for which leads to inaccurate inferences if there are many extinct lineages, 2) diversification rate estimates are highly sensitive to the prior number of rate shifts, and 3) rate estimates are unreliable because of an incorrect likelihood function. Rabosky et al.[94] subsequently demonstrated that in most cases these criticisms are incorrect, or irrelevant in biological datasets.

Nonetheless, we took steps to ensure that our lineage specific diversification rate estimates were not jeopardised by these issues. Firstly, we used two different priors for the expected number of rate shifts that differed by an order of magnitude (10 and 100). With both priors our results were highly similar (Fig. 4; Supplementary Fig. 14). Alongside this, we also estimated lineage specific diversification rates in RevBayes[81] using the dnCDBDP function. This approach maps different diversification rates as stochastic characters, differing substantially from the approach used by BAMM[96]. Diversification rates estimated in RevBayes were generally similar to those estimated by BAMM, although the magnitude and number of rate shifts was smaller (Supplementary Figs. 15-23).

Supplementary results and discussion

Here, we provide further discussion of two sets of results, the details of which underpin a number of more general findings and discussion points within the main text of the paper.

Time-dependent diversification rate estimation

Diversification rates estimated in our study from models incorporating speciation rate variation or extinction rate variation mirror each other closely. Therefore, time periods associated with higher speciation rates in the speciation variable model tend to have lower extinction rates in the extinction variable model, and *vice versa* (Supplementary Fig. 13). As a result, net diversification rate estimates from both models exhibit a very similar pattern (Supplementary Fig. 13). Nonetheless, there are important differences. In particular, higher net diversification rates (and lower extinction rates) in the extinction variable model tend to occur during slightly later time intervals than higher net diversification rates (and higher speciation rates) in the speciation variable model. This likely occurs because extinction rate shifts during one time interval affect the survival probability of lineages originating in earlier time intervals (and thus the length of surviving branches during this earlier interval). By contrast speciation rate shifts affect the rate of lineage branching during the time interval in which the speciation rate shift occurs (and thus the length of branches during that time interval). Therefore, if there is a time interval with shorter branches in the time-calibrated phylogeny (such as the time interval incorporating early branching events of the time-calibrated phylogenies estimated here) these shorter branches can be explained by a drop in the extinction rate during a subsequent time interval.

Bayes factor comparison showed that both models with variable rates were supported overwhelmingly compared to the model with constant rates (Supplementary Tab. 8). Further, these comparisons suggest that the extinction variable model is favoured over the speciation variable model. However, we are not confident in the assertion that the extinction variable model is a better reflection of the evolutionary history of angiosperms. The versions of the models we implemented for this comparison were simplified substantially (compared to the models used in our more exploratory analyses; see Supplementary Fig. 13) for computational tractability, and given the findings of Louca and Pennell[82], we consider it likely that there are alternative configurations for speciation variable models that fit the data equally as well as (or better than) the extinction variable model. Clearly, variable speciation *and* extinction

rates are likely to have occurred over the course of angiosperm evolution, although on viewing these patterns of diversification in their broader biological context, we tentatively suggest that variable speciation rates played a more important role in driving them. Below we outline the nature of this broader context and how it has helped to guide our interpretation of time-dependent diversification rate estimates.

Firstly, our analysis of gene tree conflict provides an important context to our more general conclusions about the temporal dynamics of net diversification rates in angiosperms. This is because gene tree conflict shows a temporal pattern that is highly consistent with our estimates of net diversification rates (Fig. 3), and our simulations clearly illustrate the tight association between diversification and gene tree conflict. Therefore, the temporal congruence between conflict and net diversification rates supports our net diversification rate estimates. Aside from gene tree conflict, in our analysis of lineage specific diversification rate shifts (which may be less affected by the identifiability issues[91] recently outlined by Louca and Pennell[82]) the large net diversification rate increases that occur during the Cenozoic are underpinned by *speciation* rate increases (Supplementary Fig. 24). This indicates the general increase in net diversification rates during this period may therefore be driven by lineage specific speciation rate increases. Finally, with respect to the early burst of diversification, it is notable that ~95% of all extant angiosperm orders originate during this period. Whilst conceivable that the rapid establishment of orders somehow resulted from a drop in the extinction rate, it is perhaps more intuitive to suggest that orders established rapidly during this period as a result of elevated speciation rates.

Clarifying the relationship between diversification and gene tree conflict

The simulations we undertook to analyse the relationship between gene tree conflict and time-varying speciation and extinction rates broadly supported our conclusion that conflict and diversification are tightly linked (Extended Fig. 4). However, some key nuances about this relationship were revealed. First, these simulations showed that where shifts in the net diversification rate result from speciation rate shifts,

gene tree conflict resulting from incomplete lineage sorting is directly associated with changes to the speciation rate (Extended Fig. 4). By contrast, where shifts in the net diversification rate result from changes to the extinction rate, the relationship is less direct (note the delay between increased conflict and reduced rates of extinction in Extended Fig. 4). This pattern is explained by the fact that extinction rate shifts affect branch lengths during earlier time intervals (as covered in the discussion of time-dependent diversification rate estimates) – and that under the expectation of a multi-species coalescent process, there will be more gene tree conflict associated with shorter branches in the species tree.

Ostensibly, this difference in the relationship between conflict and speciation compared to conflict and extinction could make it appear that conflict could be used as a basis for distinguishing between competing models with variable extinction and/or speciation rates. However, we do not consider this to be the case. Instead, the difference in the relationship between conflict and speciation, compared to conflict and extinction reflects the different effect that speciation and extinction have on branch lengths in the species tree. Conflict will typically be associated with regions of the species tree that have shorter branches, but these shorter branches can be induced by a myriad of time-varying speciation and extinction rates.

Additional references

85. Brown, J. W. & Smith, S. A. The Past Sure is Tense: On Interpreting Phylogenetic Divergence Time Estimates. *Syst. Biol.* **67**, 340–353 (2018).

86. Duchêne, S., Lanfear, R. & Ho, S. Y. W. The impact of calibration and clock-model choice on molecular estimates of divergence times. *Mol. Phylogenet. Evol.* **78**, 277–289 (2014).

87. Carruthers, T. & Scotland, R. W. The Implications of Interrelated Assumptions on

Estimates of Divergence Times and Rates of Diversification. *Syst. Biol.* **70**, 1181–1199 (2021).

88. Mendes, F. K. & Hahn, M. W. Gene Tree Discordance Causes Apparent Substitution Rate Variation. *Syst. Biol.* **65**, 711–721 (2016).
89. Magallón, S., Hilu, K. W. & Quandt, D. Land plant evolutionary timeline: Gene effects are secondary to fossil constraints in relaxed clock estimation of age and substitution rates. *Am. J. Bot.* **100**, 556–573 (2013).
90. Carruthers, T., Sanderson, M. J. & Scotland, R. W. The Implications of Lineage-Specific Rates for Divergence Time Estimation. *Syst. Biol.* **69**, 660–670 (2020).
91. O'Meara, B. & Beaulieu, J. Potential survival of some, but not all, diversification methods. (2021).
92. Morlon, H., Robin, S. & Hartig, F. Studying speciation and extinction dynamics from phylogenies: addressing identifiability issues. *Trends Ecol. Evol.* **37**, 497–506 (2022).
93. Moore, B. R., Höhna, S., May, M. R., Rannala, B. & Huelsenbeck, J. P. Critically evaluating the theory and performance of Bayesian analysis of macroevolutionary mixtures. *Proc. Natl. Acad. Sci.* **113**, 9569–9574 (2016).
94. Rabosky, D. L., Mitchell, J. S. & Chang, J. Is BAMM Flawed? Theoretical and Practical Concerns in the Analysis of Multi-Rate Diversification Models. *Syst. Biol.* **66**, 477–498 (2017).
95. Kubo, T. & Iwasa, Y. Inferring the rates of branching and extinction from molecular phylogenies. *Evolution* **49**, 694–704 (1995).

96. Höhna, S. *et al.* A Bayesian Approach for Estimating Branch-Specific Speciation and Extinction Rates. 555805 Preprint at https://doi.org/10.1101/555805 (2019).

Supplementary figures

High-resolution versions of all figures are available in Zenodo (https://doi.org/10.5281/zenodo.10778206).

Supplementary Fig. 1

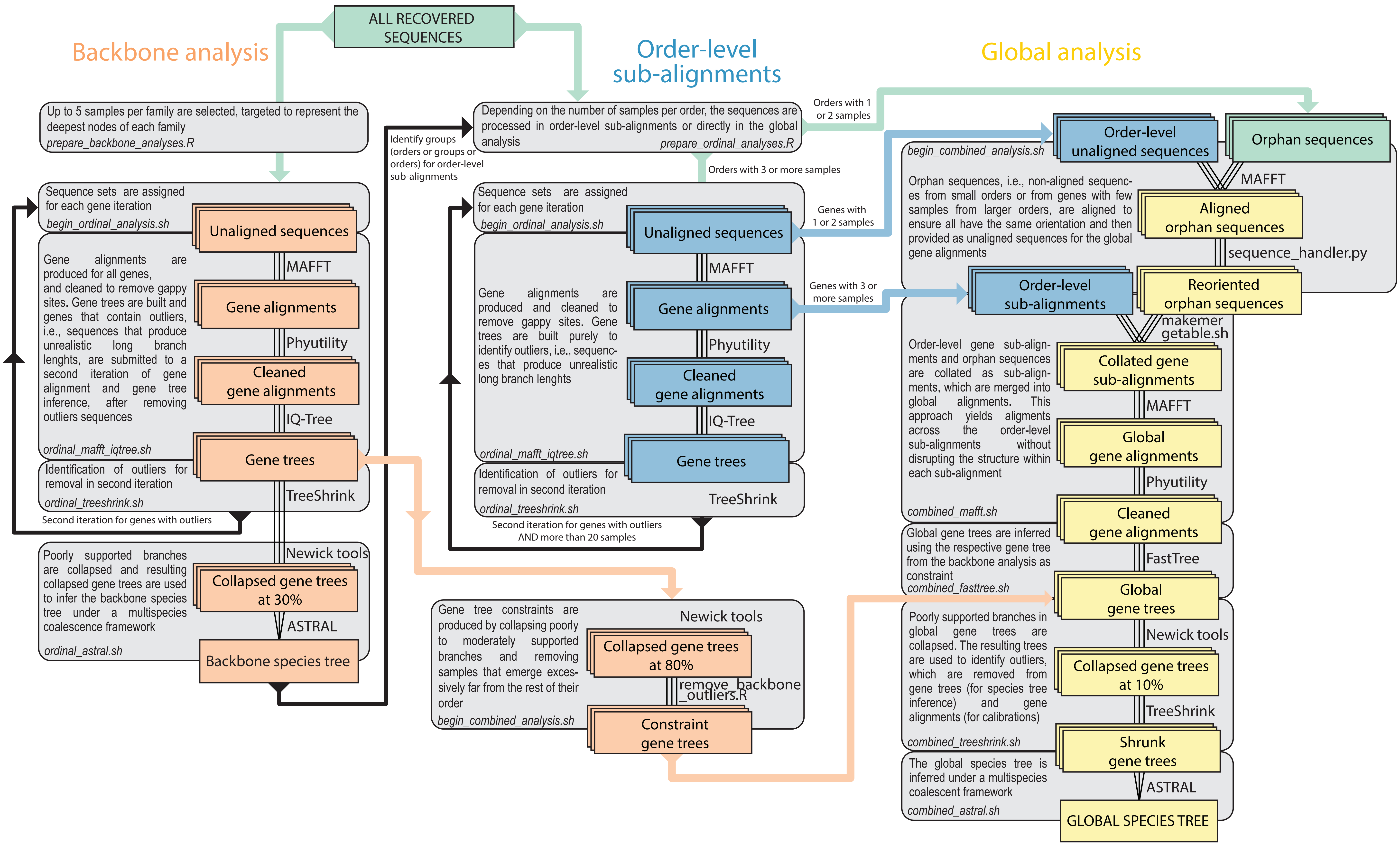

Supplementary Fig. 1 | Workflow diagram of phylogenetic inference pipeline. Small boxes represent the objects produced throughout the pipeline, coloured according to their location in the pipeline as follows: sequence recovery (green), backbone tree inference (orange), order-level sub-alignments (blue) and global tree inference (yellow). Stacked boxes represent objects handled gene-by-gene. Larger grey boxes represent analyses governed by individual scripts (script names in italics). Objects that straddle box margins are outputs of one script and inputs of another. Coloured lines represent objects handled across different sections of the pipeline. Thick black lines represent the flow of information across the pipeline.

Supplementary Fig. 2

https://doi.org/10.5281/zenodo.10778206

Supplementary Fig. 2 | Backbone species tree. Multispecies coalescent phylogenetic tree resulting from the backbone analysis. Node supported (in blue) is indicated as “posterior probability / percentage of genes supporting main hypothesis”.

Supplementary Fig. 3

https://doi.org/10.5281/zenodo.10778206

Supplementary Fig. 3 | Global species tree. Multispecies coalescent phylogenetic tree resulting from the global analysis, sampling up to five species per family. Node supported (in blue) is indicated as “posterior probability / percentage of genes supporting main hypothesis”. For a summary of relationships among deeper nodes, please refer to Extended Fig. 1 and Supplementary Fig. 4.

Supplementary Fig. 4

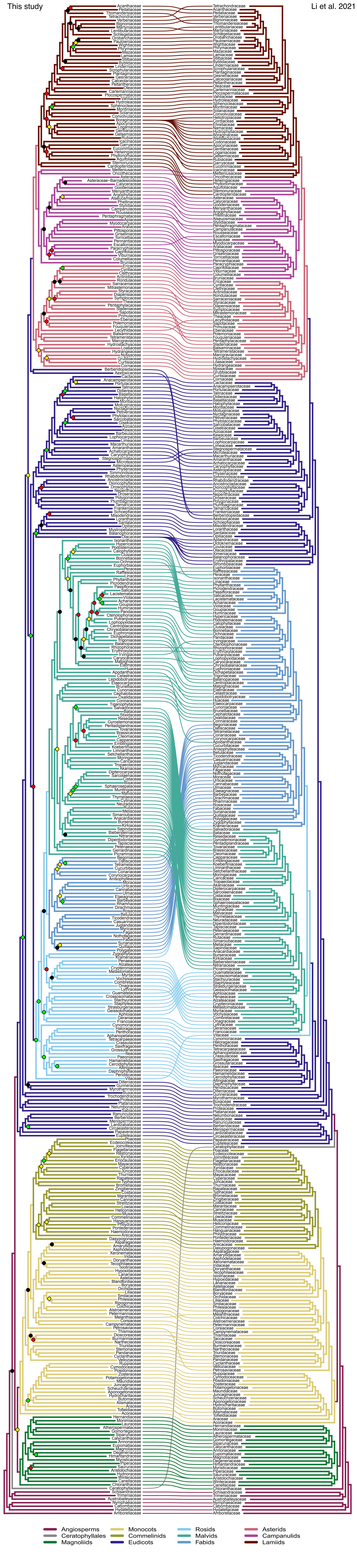

Supplementary Fig. 4 | Tanglegram comparing relationships among angiosperm families in this study with Li et al. (2021). Branch colours indicate the major clades to which each family belongs, according to the circumscriptions adopted in our study (left) and Li et al.[4] (right), which is broadly consistent with APG IV[18]. Coloured circles in the tree (left) represent the posterior probability of each node as: maximum (absent), between 1 and 0.95 (green), between 0.95 and 0.75 (yellow), between 0.75 and 0.5 (red), below 0.5 (black)

Supplementary Fig. 5

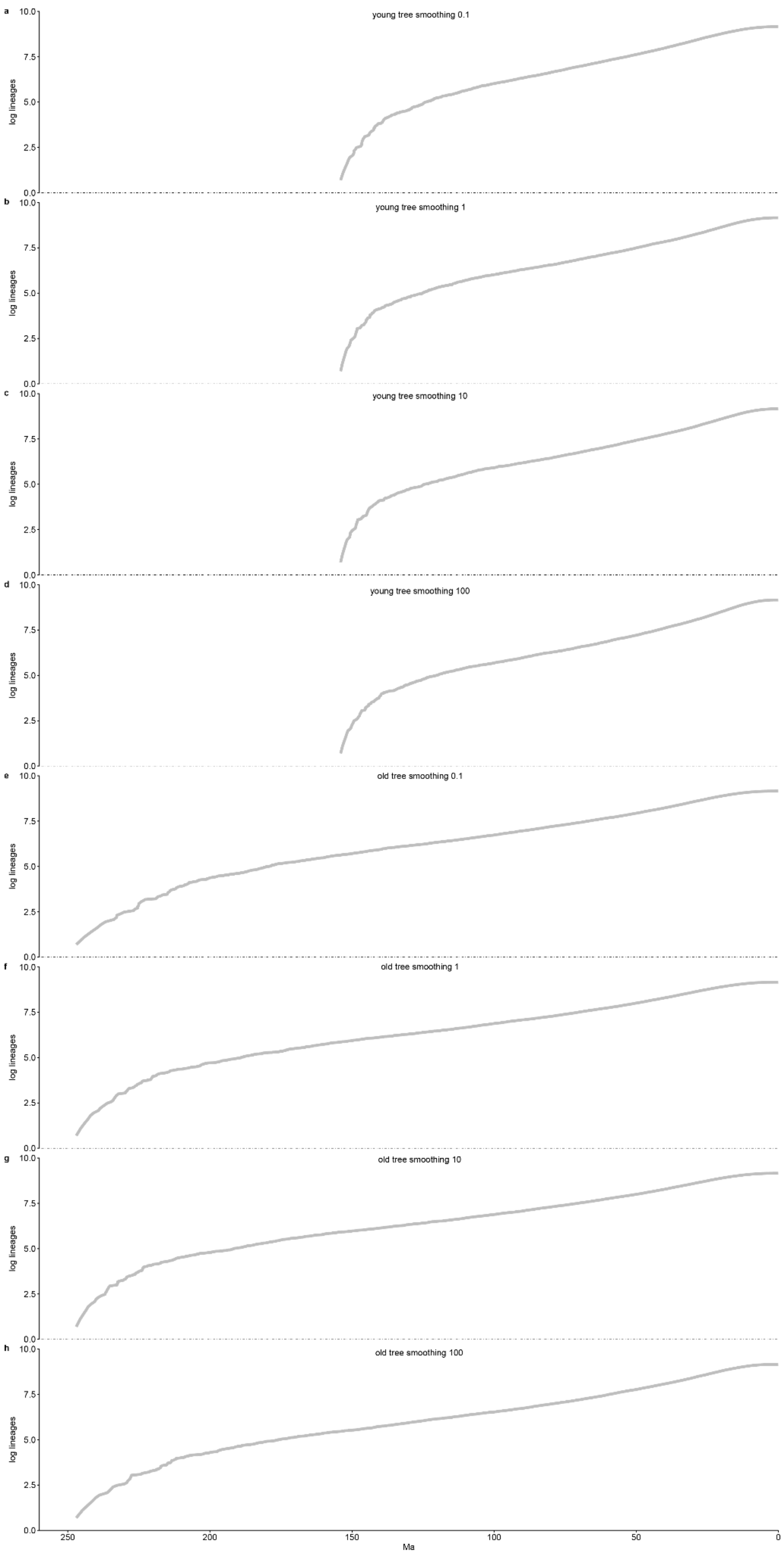

Supplementary Fig. 5 | Lineage through time plots for the eight time-calibrated phylogenetic trees. Comparison of the shape of the plots gives an indication of the temporal distribution of node ages in each time-calibrated phylogenetic tree.

Supplementary Fig. 6

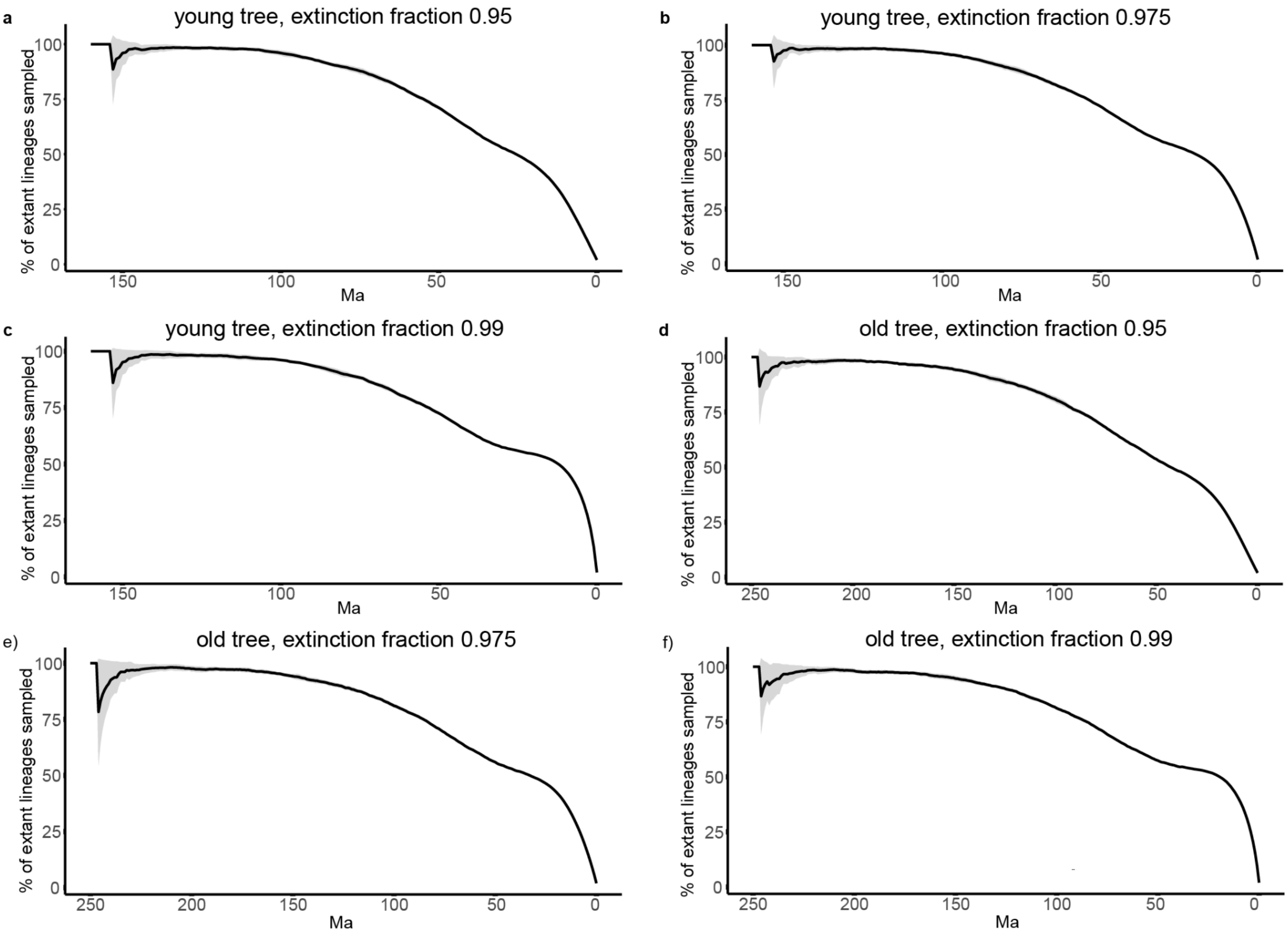

Supplementary Fig. 6 | Percentage of extant lineages sampled through time. The results are based on the young tree (maximum constraint at the root node of 154Ma) and old tree (maximum constraint at the root node of 247Ma), as predicted by our simulation-based approach. The black line is the mean estimate across replicate simulations, the grey shaded area is two standard deviations around the mean. In a series of analyses, different extinction fractions were specified (as indicated), which affects the shape of the curve, particularly in more recent times.

Supplementary Fig. 7

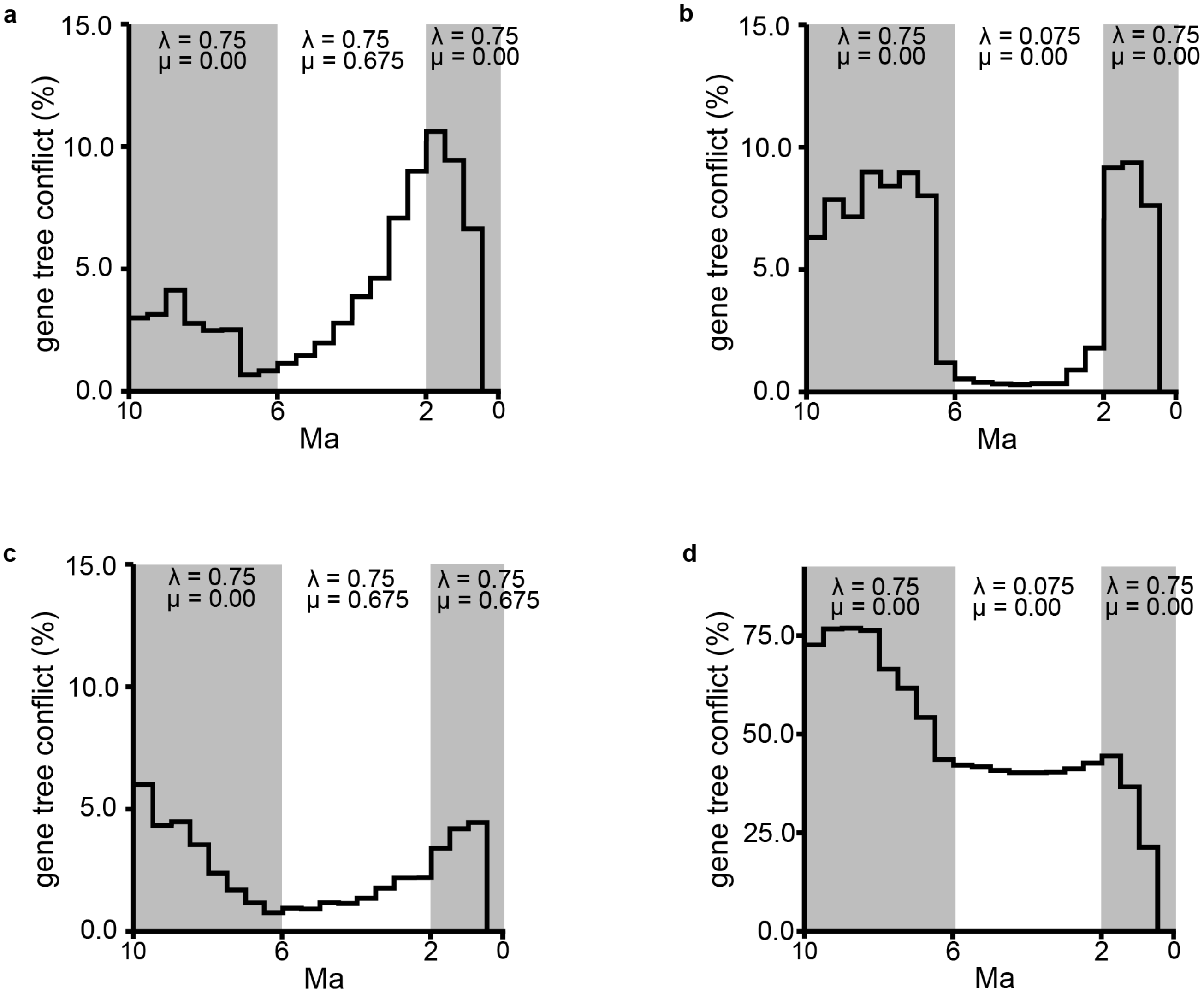

Supplementary Fig. 7 | Simulations exploring the relationship between gene tree conflict and diversification. The line in each plot shows the level of gene tree conflict through time. This is calculated as the percentage of gene trees that do not share a congruent bipartition with each species tree branch, with the plotted value being the mean across all species tree branches that cross each 0.5 Myr time slice. Species trees are simulated under different speciation rate and extinction rate parameters in each experiment. **a-c,** Have an effective population size of 5000, **d,** Has an effective population size of 50000.

Supplementary Fig. 8

https://doi.org/10.5281/zenodo.10778206

Supplementary Fig. 8 | Gene tree conflict across the phylogeny. The results are based on the young tree (maximum constraint at the root node of 154Ma). Branch colours represent the percentage of gene trees that do not share a congruent bipartition with the species tree branch.

Supplementary Fig. 9

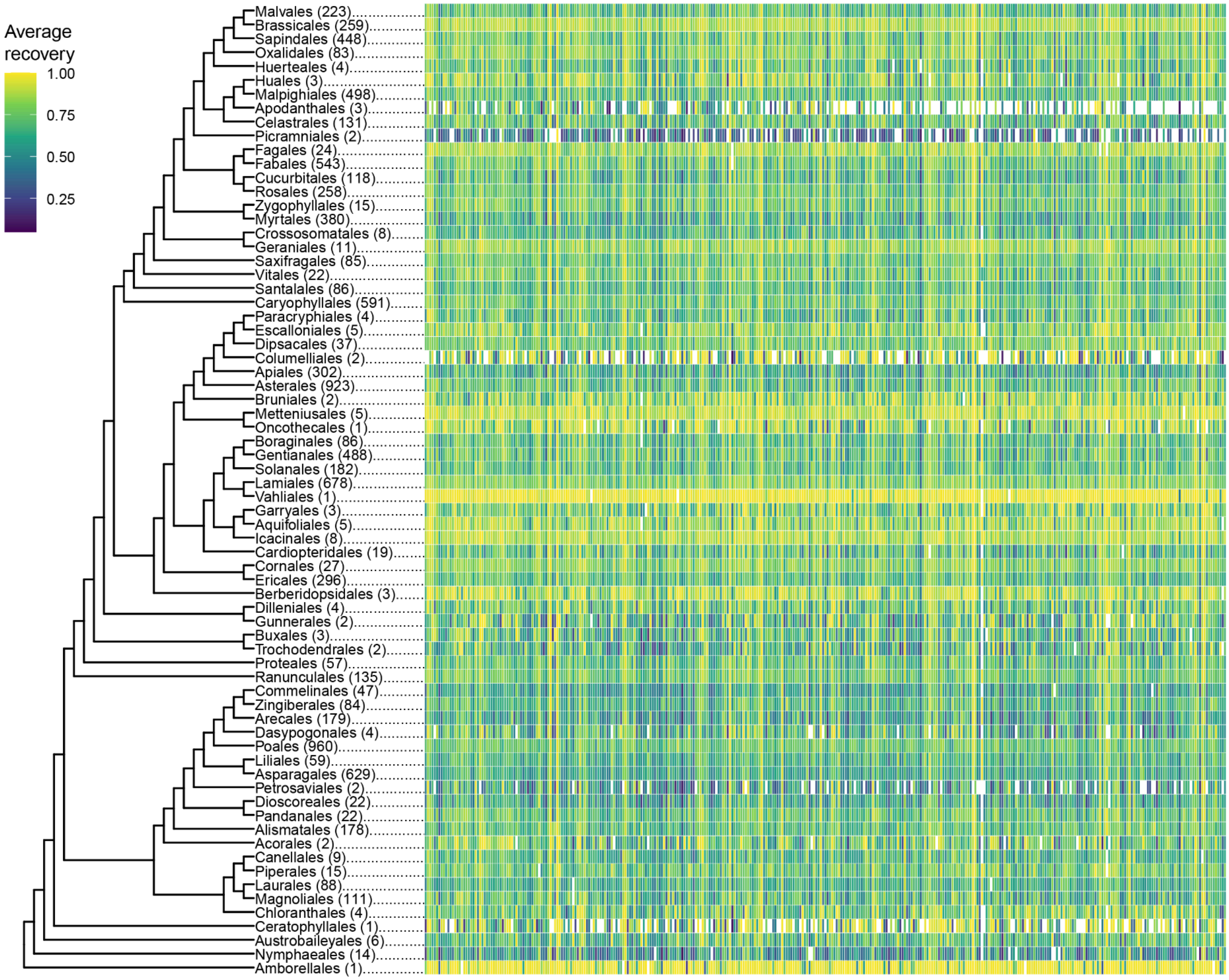

Supplementary Fig. 9 | Heatmap of relative gene recovery per order. Columns within the heatmap represent genes. The expected length of each gene was calculated as the average length of all sequences in the target file for each gene. Numbers within parentheses next to orders indicate the number of samples for that group. Blank cells indicate zero recovery.

Supplementary Fig. 10

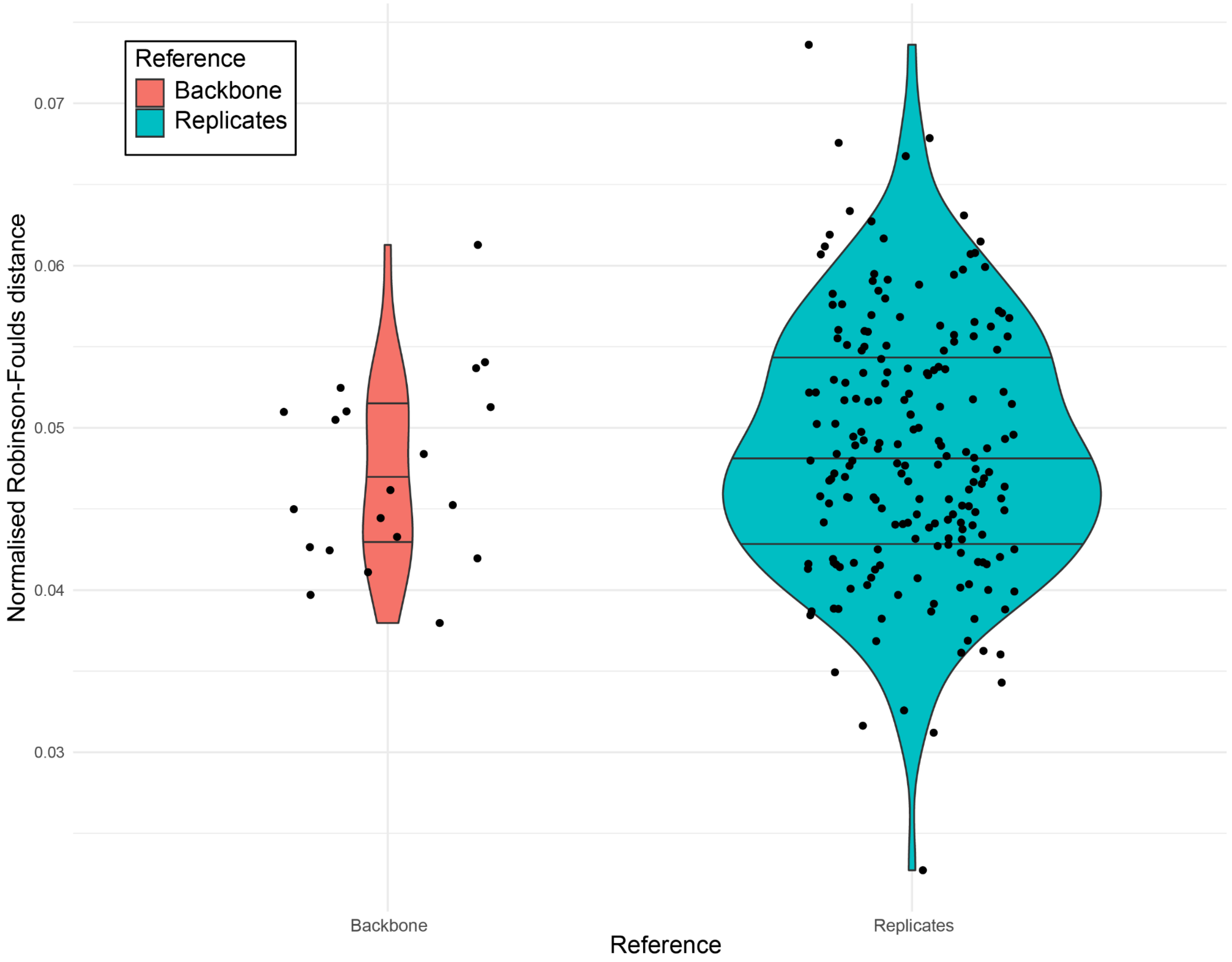

Supplementary Fig. 10 | Pairwise comparison of normalised Robinson-Foulds distances between the backbone tree and replicates. Distances to the backbone tree (left; mean: 4.71%) are not significantly different (p-value = 0.3127 in two-tailed T-test) to the distances among replicates (right; mean: 4.86%). This result indicates that our sample selection in the backbone tree does not introduce any systemic bias to the phylogenetic inference.

Supplementary Fig. 11

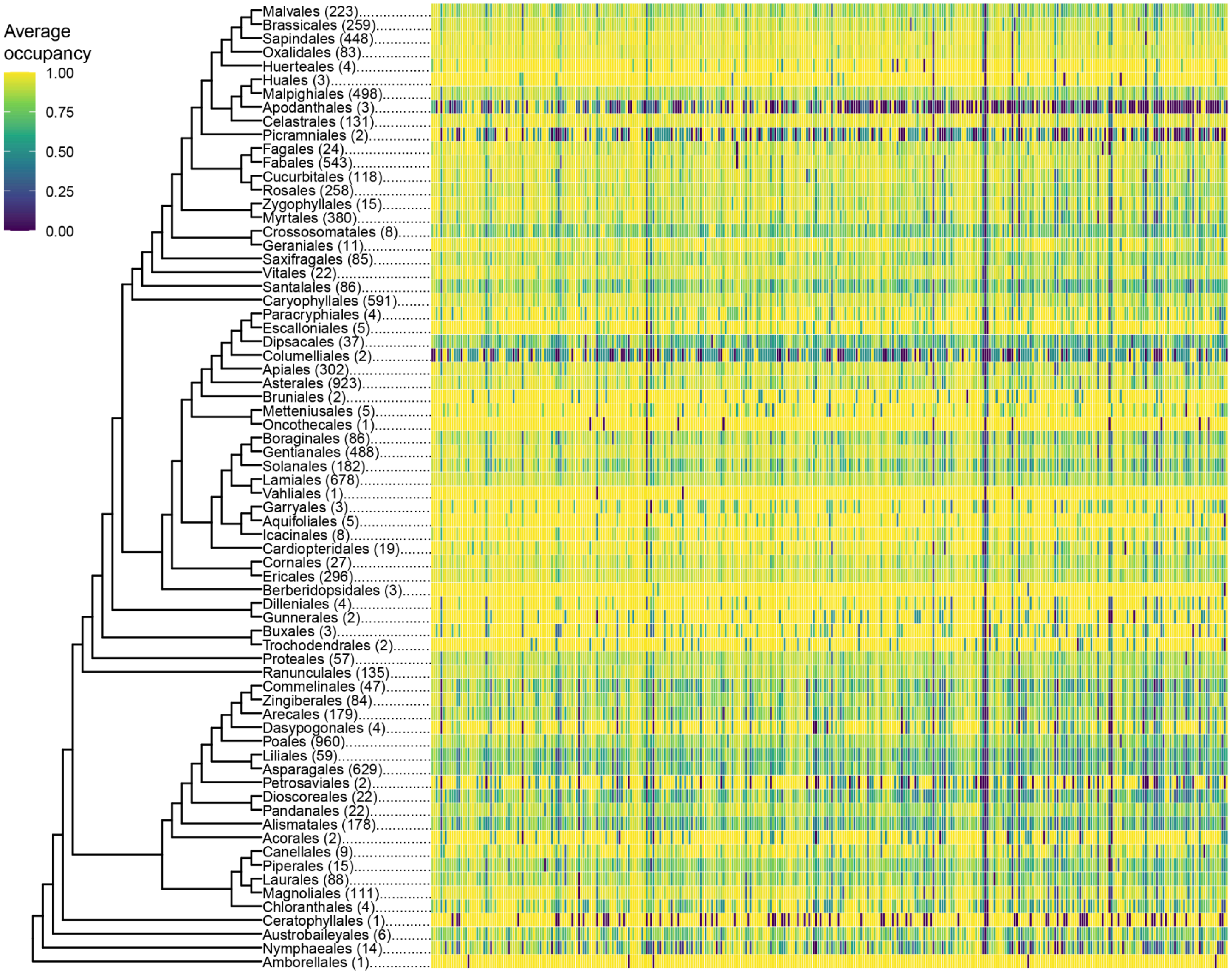

Supplementary Fig. 11 | Heatmap of gene occupancy in alignments per order. Columns within the heatmap represent genes. Occupancy is calculated as the proportion of samples per order for which any data were retained in each gene alignment. Numbers within parentheses next to orders indicate the number of samples for that group.

Supplementary Fig. 12

https://doi.org/10.5281/zenodo.10778206

Supplementary Fig. 12 | Phylogenetic distribution of fossil calibrations. At each numbered node a fossil calibration has been used. The number refers to the ID number in AngioCal V1.1 (Supplementary Tab. 6). This also corresponds to column 5 in Supplementary Tab. 7.

Supplementary Fig. 13

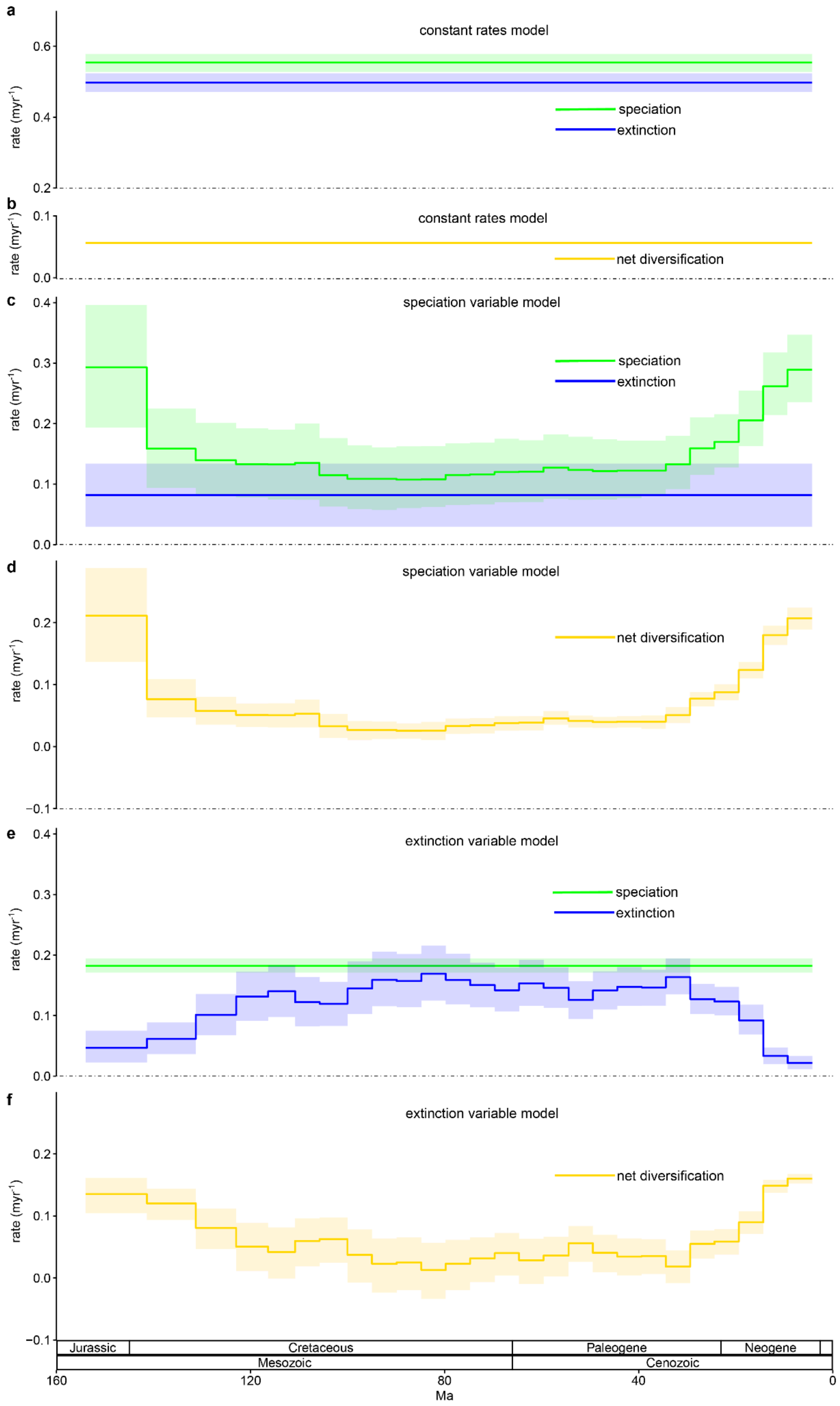

Supplementary Fig. 13 | Comparison of speciation rate, extinction rate, and net diversification rate estimates from different time dependent diversification rate models that were used in this study. In each case, the lines refer to the posterior mean and the shaded area around each line refers to the 95% HPD. **a,** and **b,** Show the estimated speciation rate, extinction rate and net diversification rate through time in the constant rates model. **c,** and **d,** Show the estimated speciation rate, extinction rate, and net diversification rate through time in the speciation variable model. **e,** and **f,** Show the estimated speciation rate, extinction rate, and net diversification rate through time in the extinction variable model.

Supplementary Fig. 14

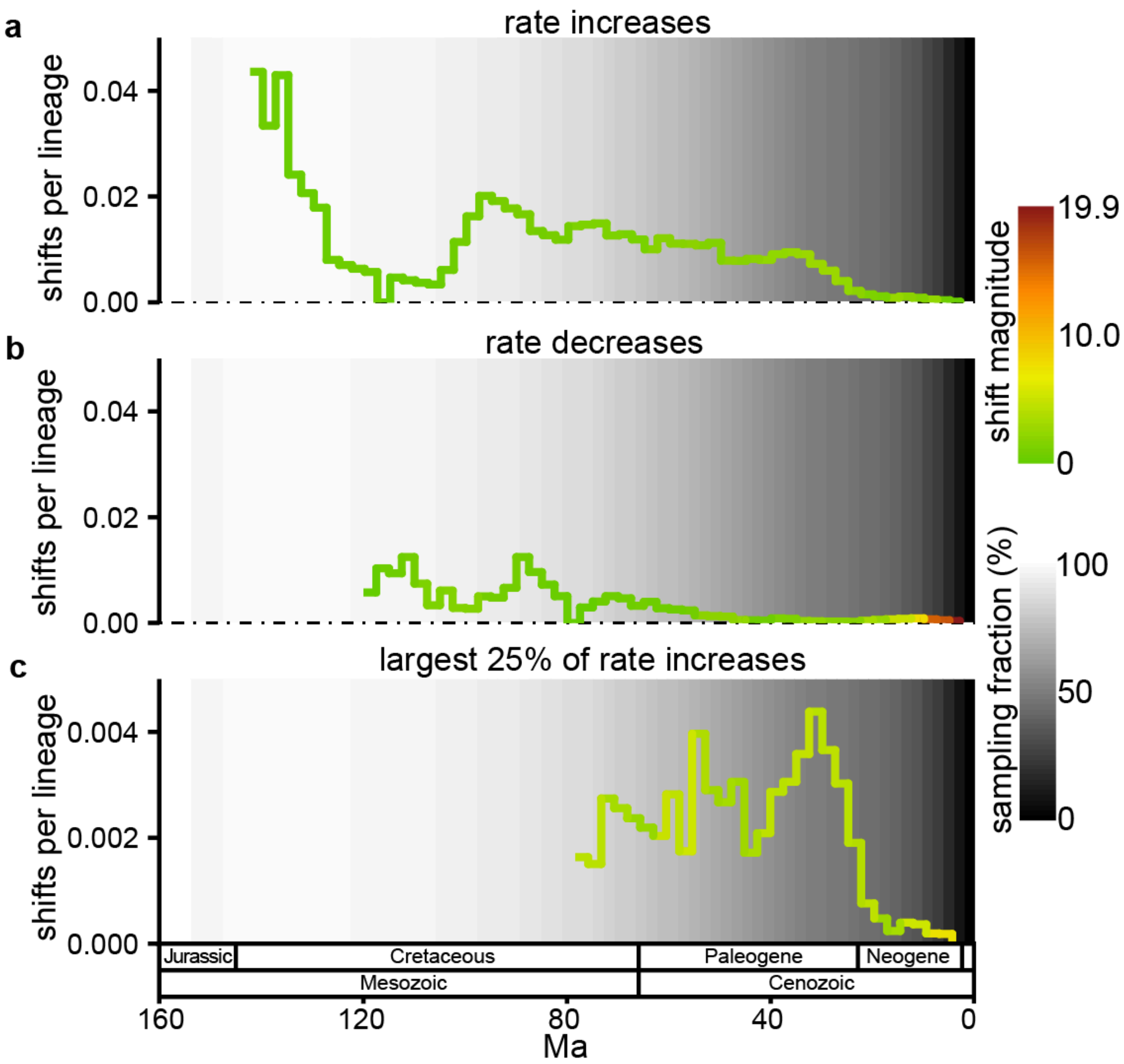

Supplementary Fig. 14 | Summary of lineage-specific diversification rate shifts estimated by BAMM for the young tree (maximum constraint at the root node of 154 Ma). This is equivalent to Figure 4 but with the prior for the number of shifts set to 100. **a**, Diversification rate increases per lineage through time. The colour corresponds to the average magnitude of the rate increases during the time period. **b**, Equivalent to a**,** but for rate decreases. **c**, Equivalent to a, but focusing on the largest 25% of diversification rate increases. In **a**, **b**, and **c**, the number of shifts is extracted from the maximum a posteriori shift configuration, and the background grey-scale gradient is the estimated percentage of extant lineages represented in the species tree through time ("sampling fraction").

Supplementary Fig. 15

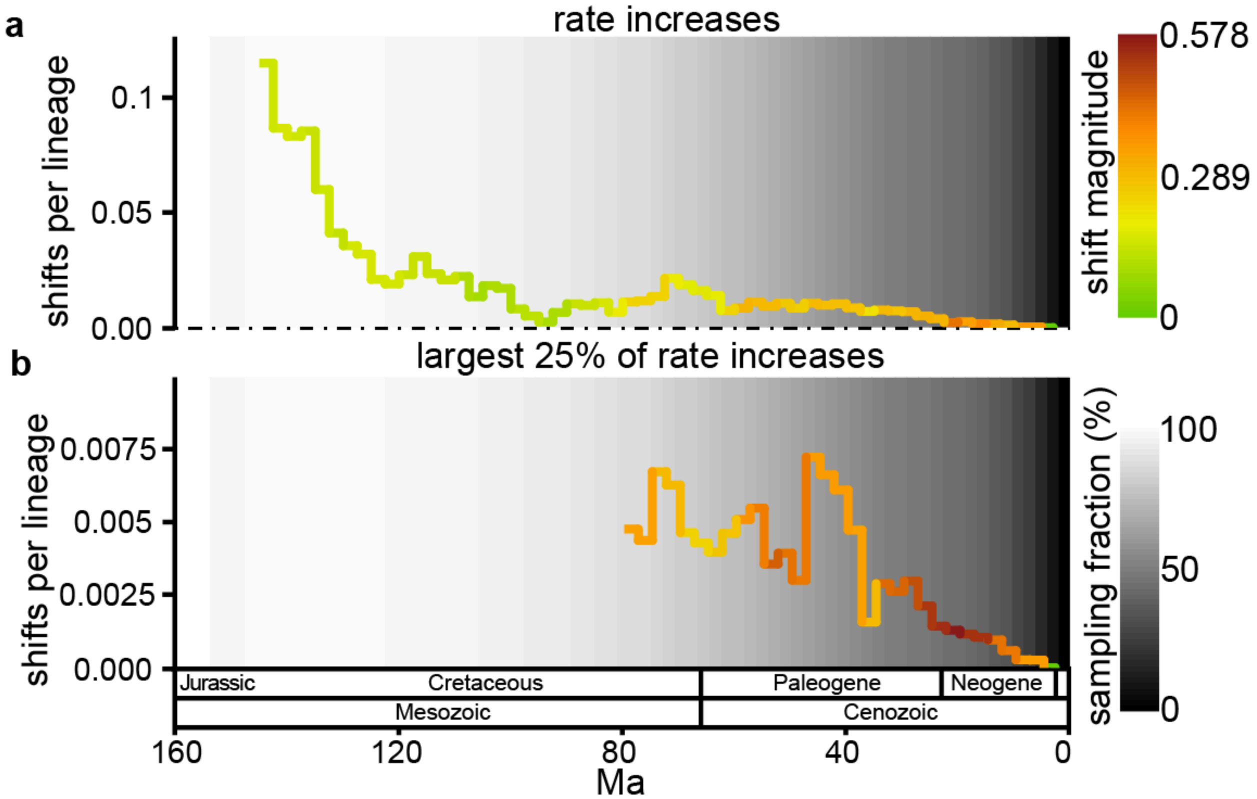

Supplementary Fig. 15 | Summary of lineage-specific diversification rate shifts estimated by RevBayes for the young tree (maximum constraint at the root node of 154 Ma). The number of shifts through time is derived from the mean posterior rate estimate for each branch, with rate shifts being defined as cases where there is a greater than 10% rate difference between ancestral and descendant branches. Unlike with analyses in BAMM, shifts are not derived from a maximum a posteriori shift configuration because several shift configurations had an equal posterior probability. **a**, Diversification rate increases per lineage through time. The colour corresponds to the average magnitude of the rate increases during the time period. **b**, Equivalent to a, but focusing on the largest 25% of diversification rate increases. There is no panel for rate decreases because RevBayes only estimated small rate decreases (< 10% between ancestral and descendant branches in all cases). In **a** and **b** the prior for the number of shifts is set to 100, and the background grey-scale gradient is the estimated percentage of extant lineages represented in the species tree through time (“sampling fraction”).

Supplementary Fig. 16

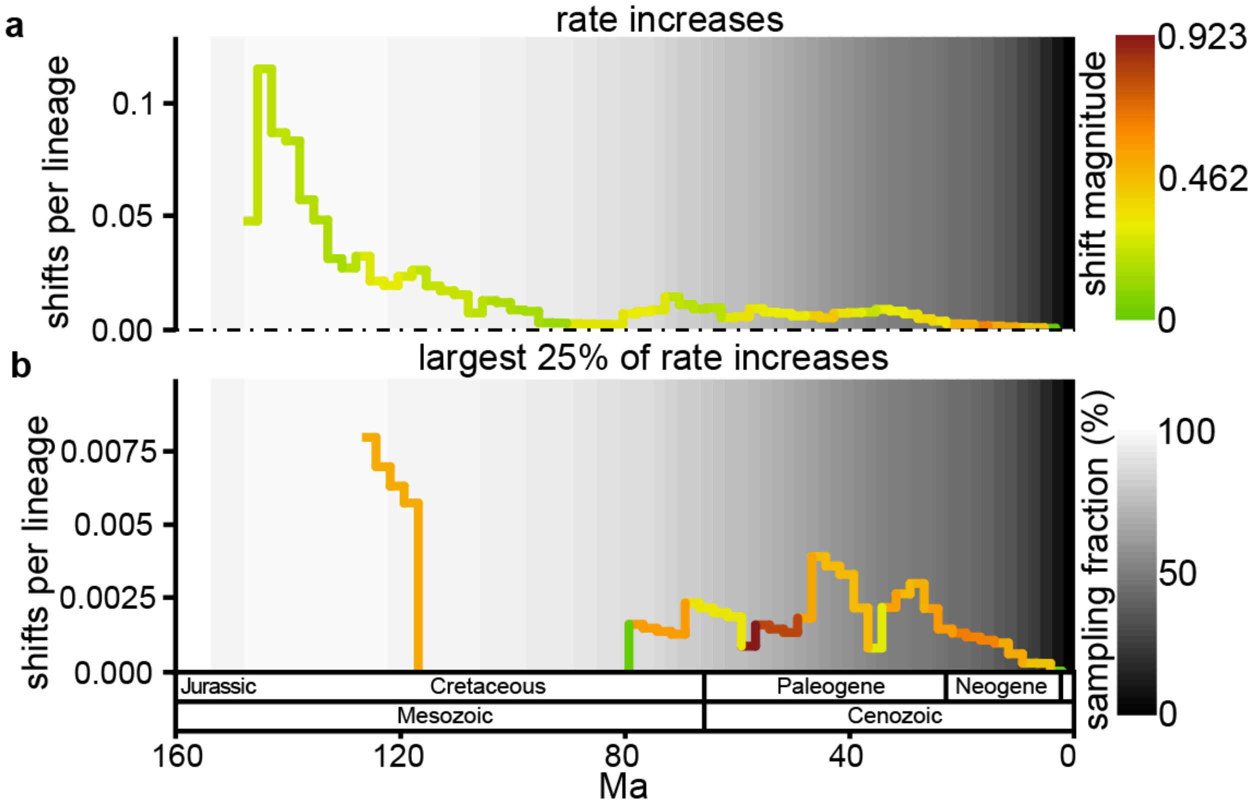

Supplementary Fig. 16 | Summary of lineage-specific diversification rate shifts estimated by RevBayes for the young tree (maximum constraint at the root node of 154 Ma). The number of shifts through time is derived from the mean posterior rate estimate for each branch, with rate shifts being defined as cases where there is a greater than 10% rate difference between ancestral and descendant branches. Unlike with analyses in BAMM, shifts are not derived from a maximum a posteriori shift configuration because several shift configurations had an equal posterior probability. **a**, Diversification rate increases per lineage through time. The colour corresponds to the average magnitude of the rate increases during the time period. **b**, Equivalent to a, but focusing on the largest 25% of diversification rate increases. There is no panel for rate decreases because RevBayes only estimated small rate decreases (< 10% between ancestral and descendant branches in all cases). In **a** and **b** the prior for the number of shifts is set to 10, and the background grey-scale gradient is the estimated percentage of extant lineages represented in the species tree through time (“sampling fraction”).

Supplementary Fig. 17

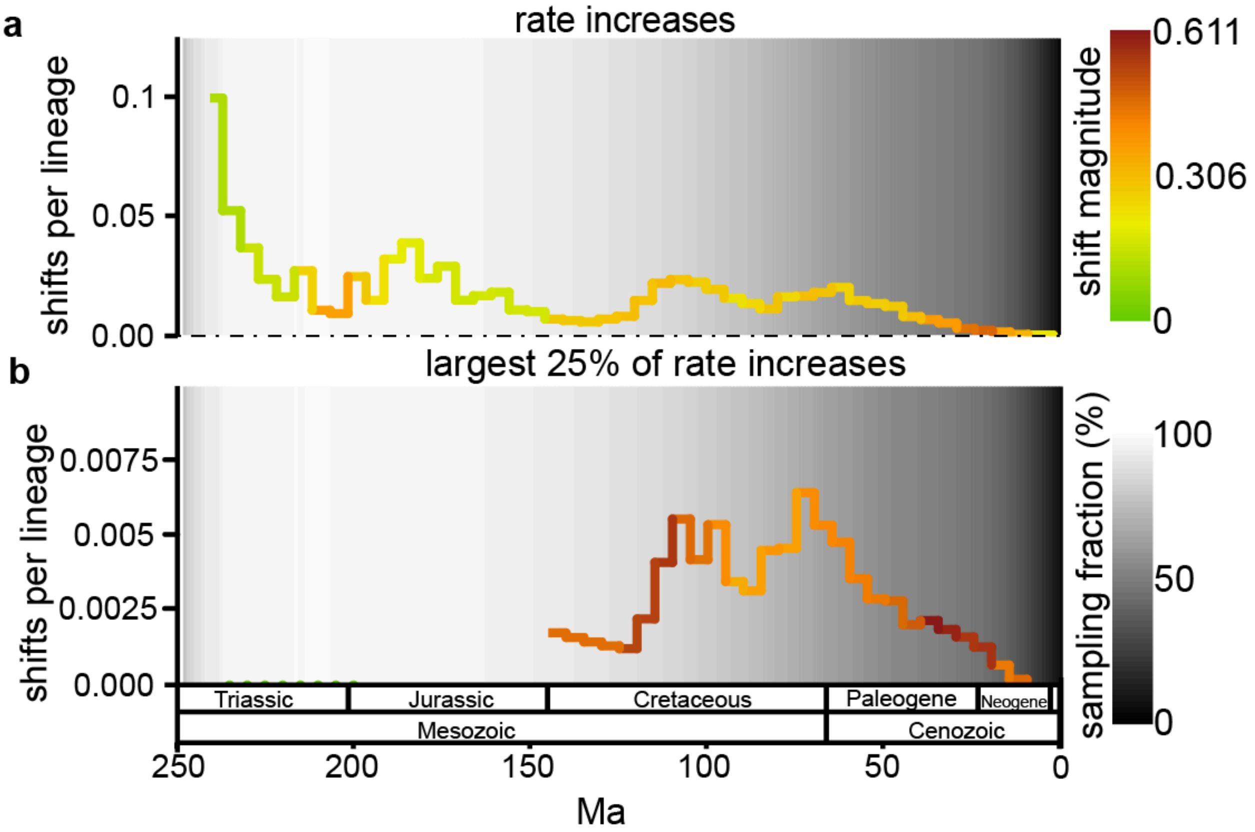

Supplementary Fig. 17 | Summary of lineage-specific diversification rate shifts estimated by RevBayes for the old tree (maximum constraint at the root node of 247 Ma). The number of shifts through time is derived from the mean posterior rate estimate for each branch, with rate shifts being defined as cases where there is a greater than 10% rate difference between ancestral and descendant branches. Unlike with analyses in BAMM, shifts are not derived from a maximum a posteriori shift configuration because several shift configurations had an equal posterior probability. **a**, Diversification rate increases per lineage through time. The colour corresponds to the average magnitude of the rate increases during the time period. **b**, Equivalent to a, but focusing on the largest 25% of diversification rate increases. There is no panel for rate decreases because RevBayes only estimated small rate decreases (< 10% between ancestral and descendant branches in all cases). In **a** and **b** the prior for the number of shifts is set to 100, and the background grey-scale gradient is the estimated percentage of extant lineages represented in the species tree through time (“sampling fraction”).

Supplementary Fig. 18

https://doi.org/10.5281/zenodo.10778206

Supplementary Fig. 18 | Net diversification rates estimated in BAMM for the young tree (maximum constraint at the root node of 154 Ma). The plotted shift configuration represents the configuration with the maximum a posteriori probability, with branch colours representing the net diversification rate. The prior for the number of shifts is set to 10.

Supplementary Fig. 19

https://doi.org/10.5281/zenodo.10778206

Supplementary Fig. 19 | Net diversification rates estimated in BAMM for the old tree (maximum constraint at the root node of 247 Ma). The plotted shift configuration represents the configuration with the maximum a posteriori probability, with branch colours representing the net diversification rate. The prior for the number of shifts is set to 10.

Supplementary Fig. 20

https://doi.org/10.5281/zenodo.10778206

Supplementary Fig. 20 | Net diversification rates estimated in BAMM for the young tree (maximum constraint at the root node of 154 Ma). The plotted shift configuration represents the configuration with the maximum a posteriori probability, with branch colours representing the net diversification rate. The prior for the number of shifts is set to 100.

Supplementary Fig. 21

https://doi.org/10.5281/zenodo.10778206

Supplementary Fig. 21 | Net diversification rates estimated in RevBayes for the young tree (maximum constraint at the root node of 154 Ma). The plotted rates are the mean posterior rate estimate for each branch, with branch colours representing the net diversification rate. The prior for the number of shifts is set to 100.

Supplementary Fig. 22

https://doi.org/10.5281/zenodo.10778206

Supplementary Fig. 22 | Net diversification rates estimated in RevBayes for the old tree (maximum constraint at the root node of 247 Ma). The plotted rates are the mean posterior rate estimate for each branch, with branch colours representing the net diversification rate. The prior for the number of shifts is set to 100.

Supplementary Fig. 23

https://doi.org/10.5281/zenodo.10778206

Supplementary Fig. 23 | Net diversification rates estimated in RevBayes for the young tree (maximum constraint at the root node of 154 Ma). The plotted rates are the mean posterior rate estimate for each branch, with branch colours representing the net diversification rate. The prior for the number of shifts is set to 10.

Supplementary Fig. 24

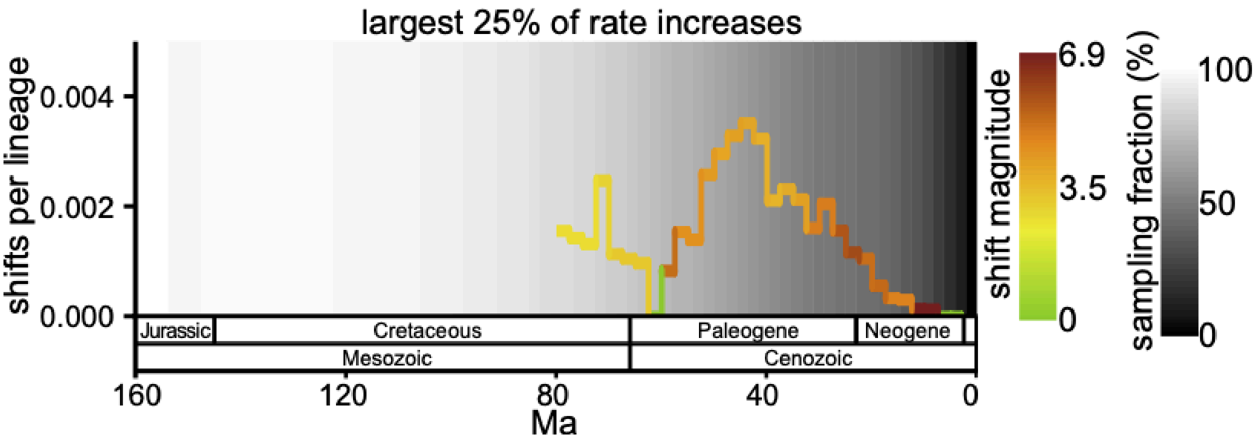

Supplementary Fig. 24 | The number of large speciation rate increases through time in the young tree (maximum constraint at the root node of 154 Ma). The number of shifts is extracted from the maximum a posteriori shift configuration, the prior for the number of shifts is set to 10, and the background grey-scale gradient is the estimated percentage of extant lineages represented in the species tree through time ("sampling fraction").

Supplementary tables

All supplementary tables are available in Zenodo (https://doi.org/10.5281/zenodo.10778206).

Supplementary Tab. 1 | Sample metadata and recovery statistics. Metadata is presented for all samples included in this work, including data source, voucher information for data generated for this study, ENA/DDBJ/NCBI accession numbers, recovery statistics, occupancy in Astral analysis, groups for order-level sub-alignments and the presence of each sample in the backbone, calibrated tree and pruned tree. Abbreviations in source column: GAP - Genomics for Australian Plants project, PAFTOL - Plant and Fungal Trees of Life Project, EVOFLORAND - Biogeography, evolution, ecology and conservation of the Andalusian flora, OneKP - One Thousand Plant Transcriptomes Initiative[9], SRA - Sequence Read Archive.

Supplementary Tab. 2 | Details of plant portraits illustrating Fig. 1. All plant portraits were obtained from Curtis's Botanical Magazine (via the Biodiversity Heritage Library). For each plant portrait, the artist, volume and year of publication are provided, followed by Biodiversity Heritage Library (BHL) unique page id, and both originally published name and currently accepted name. Portraits are ordered clockwise, starting from the ANA Grade.

Supplementary Tab. 3 | Ages of major clades, orders and families. Ages of stem and crown nodes are provided from both young and old trees (maximum constraint at the root node 154 Ma and 247 Ma, respectively). For each clade, the number of samples within that clade and the node number of the clade in the calibrated tree file is provided.

Supplementary Tab. 4 | Number of rate shifts per order. It includes the number of overall and nested shifts. The results are based on the young tree (maximum constraint at the root node of 154 Ma).

Supplementary Tab. 5 | Gene alignments summary and SortaDate results. A summary of each combined gene alignment (after cleaning) is reported, showing dimensions and composition of each. Alignment summaries were produced using AMAS[71]. The genes are sorted according to the SortaDate ranking.

Supplementary Tab. 6 | Fossil calibration dataset – AngioCal v1.1. Updated version of the fossil calibration dataset, AngioCal v1.0, from Ramírez-Barahona et al.[5]

Supplementary Tab. 7 | Fossil calibrations points used in this study. For each fossil, the age used and the node description are provided, followed by the tips in our tree used to define that node and the ID Number in AngioCal v1.1 (Supplementary Tab. 4). The phylogenetic placement can be observed in Supplementary Fig. 12.

Supplementary Tab. 8 | Bayes factor comparison for different time variable diversification rate models. The maximum likelihood from the three diversification models (constant rates, variable speciation and variable extinction) is presented with brief interpretation.

Supplementary files

All supplementary files are available in Zenodo (https://doi.org/10.5281/zenodo.10778206).

Supplementary File 1 | Target file used for sequence recovery. Reference sequences translated into amino acids used during the sequence recovery.

Supplementary File 2 | Fossil calibration dataset – AngioCal v1.1. Updated version of the fossil calibration dataset, AngioCal v1.0, from Ramírez-Barahona et al.[5]

Extended acknowledgements

The authors would like to acknowledge the contribution of the Genomics for Australian Plants Framework Initiative consortium (https://www.genomicsforaustralianplants.com/consortium/) in the generation of data used in this publication. The Initiative is supported by funding from Bioplatforms Australia (enabled by NCRIS), the Ian Potter Foundation, Royal Botanic Gardens Foundation (Victoria), Royal Botanic Gardens Victoria, the Royal Botanic Gardens and Domain Trust, the Council of Heads of Australasian Herbaria, CSIRO, Centre for Australian National Biodiversity Research and the Department of Biodiversity, Conservation and Attractions, Western Australia.

Juan Arroyo and Montserrat Arista would like to thank "Red de Jardines Botánicos en Espacios Protegidos" in Andalusia for facilitating the sourcing of specimens and permit provision; ERDF/FEDER from the European Union, the General Directorate for Research and Knowledge Transfer (Regional Government of Andalusia), and the University of Seville (grants BIOVEGAN P18-RT-3651 and EVOFLORAND US-1265280) for funding.

Lisa Pokorny benefited from a Ramón y Cajal grant (RYC2021-034942-I) funded by MCIN/AEI/10.13039/501100011033 and by the European Union “NextGenerationEU”/PRTR.

Guilherme Antar would like to thank CAPES and Bentham Moxon Trust for funding.

Jeremy Bruhl would like to thank Danielle Smith for curation and despatch of silica gel collections at the N.C.W. Beadle Herbarium (NE).

Anne Bruneau was funded by the Natural Sciences and Engineering Research Council of Canada (NSERC).

Paola Ferreira would like to thank Coordenação de Aperfeiçoamento de Pessoal de Nível Superior (CAPES; PDSE proc. 88881.132410/2016-01), Missouri Botanical Garden Elisabeth E. Bascom Scholarship (2017), and Fundação de Amparo à Pesquisa do Estado de São Paulo (FAPESP; 2016/06260-2) for funding.

Cynthia González, Oriane Hidalgo & Luis Palazzesi would like to thank the staff of Dirección de Fauna y Flora Silvestre de la Provincia del Chubut in Argentina for facilitating the procedures for collecting materials, and the National University of Patagonia San Juan Bosco for financing the project PI 1416 (CDFCNyCS 503/17).

Jan Hackel was funded by a Future Leader Fellowship from the Royal Botanic Gardens, Kew.

Oriane Hidalgo thanks Inés Álvarez, Santiago Andrés, Manica Balant, Nicola Bergh, Mauricio Diazgranado, Rolland Douzet, Jair E.Q. Faria, Leonardo P. Felix, Susana Freire, Federico García, Teresa Garnatje, Federico Luebert, Jordi Luque and Laura Martínez Suz for field assistance and sample supply.

Frederic Lens acknowledges the German Research Foundation (DFG; grant numbers MU1137/17-1 and KO2302/23-2).

Tatyana Livshultz would like to thank Chelsea R. Smith for laboratory assistance, David J. Middleton and Mary E. Endress for providing specimens, and the National Science Foundation (DEB-1655663) for funding.

Jaquelini Luber would like to thank National Geographic for funding the project "Phylogeny, Taxonomy and Conservation of *Neocalyptrocalyx* Hutch. (Capparaceae)".

Vincent Merckx received funding from the European Research Council (ERC) under the European Union's Horizon 2020 research and innovation programme (grant agreement No 101045057).

Rosa Isela Meneses thanks the Chilean National Agency for Research and Development, Chilean National Agency for Research and Development (ANID), (FONDECYT/DOCTORADO NACIONAL / 2020 – 21201693).

Luis Palazzesi thanks Gonzalo Nieto, Luca Pegoraro, Iván Pérez-Lorenzo, Samuel Pyke, Yannis Robert, Caetano Troncoso Oliveira, Estrella Urtubey, Joan Vallès, Thais N.C. Vasconcelos, Iralys Ventosa, Maximilian Weigend and Yannick Woudstra for field assistance and sample supply.

Julian Starr would like to thank Bruce Ford for field assistance, the herbaria at BOL, EA, GENT, K,NE, UPOS and WIN for the use of their specimens, and the National Science and Engineering Research Council (NSERC) of Canada for funding (Discovery Grant to JRS, RGPIN 2018-04115).

Cassiano A. Dorneles Welker would like to thank Conselho Nacional de Desenvolvimento Científico e Tecnológico (CNPq; 426334/2018-3, 441760/2020-1, 307125/2020-3) and Fundação de Amparo à Pesquisa do Estado de Minas Gerais (FAPEMIG; APQ-01222-21, APQ-03365-21) for funding.

Alexandre Antonelli acknowledges financial support from the Swedish Research Council (2019-05191), the Swedish Foundation for Strategic Environmental Research MISTRA (Project BioPath), and the Kew Foundation.
