# Supplementary material for: Phylogenomics and the rise of the angiosperms
Source: Nature. 2024 Apr 24;629(8013):843–50. doi: 10.1038/s41586-024-07324-0 (PMC11111409; doi:10.1038/s41586-024-07324-0)
Supplement: Supplementary file 2 — Reporting Summary [file 41586_2024_7324_MOESM2_ESM.pdf]

nature portfolio

Corresponding author(s): William J. Baker

Last updated by author(s): Mar 5, 2024

Reporting Summary

Nature Portfolio wishes to improve the reproducibility of the work that we publish. This form provides structure for consistency and transparency in reporting. For further information on Nature Portfolio policies, see our Editorial Policies and the Editorial Policy Checklist.

Statistics

For all statistical analyses, confirm that the following items are present in the figure legend, table legend, main text, or Methods section.

n/a	Confirmed	
☒	☐	The exact sample size (n) for each experimental group/condition, given as a discrete number and unit of measurement
☒	☐	A statement on whether measurements were taken from distinct samples or whether the same sample was measured repeatedly
☐	☒	The statistical test(s) used AND whether they are one- or two-sided<br>*Only common tests should be described solely by name; describe more complex techniques in the Methods section.*
☒	☐	A description of all covariates tested
☒	☐	A description of any assumptions or corrections, such as tests of normality and adjustment for multiple comparisons
☐	☒	A full description of the statistical parameters including central tendency (e.g. means) or other basic estimates (e.g. regression coefficient) AND variation (e.g. standard deviation) or associated estimates of uncertainty (e.g. confidence intervals)
☐	☒	For null hypothesis testing, the test statistic (e.g. F, t, r) with confidence intervals, effect sizes, degrees of freedom and P value noted<br>*Give P values as exact values whenever suitable.*
☐	☒	For Bayesian analysis, information on the choice of priors and Markov chain Monte Carlo settings
☒	☐	For hierarchical and complex designs, identification of the appropriate level for tests and full reporting of outcomes
☒	☐	Estimates of effect sizes (e.g. Cohen's d, Pearson's r), indicating how they were calculated

Our web collection on statistics for biologists contains articles on many of the points above.

Software and code

Policy information about availability of computer code

Data collection	No software was used.
Data analysis	Raw reads were trimmed using trimmomatic 0.39. Data recovery was performed in HybPiper 1.3.1, which includes blast 2.5.0, exonerate 2.4.0, parallel 20200922 and SPADES 3.13.0. Phylogenetic inference was performed using ASTRAL-III 5.7.3, ASTRAL-MP 5.15.5, fasttree 2.1.10, IQ-Tree 2.2.0-beta, MAFFT v7.480, newick_utils 1.6, phyutility 2.7.1, SortaDate commit 15bbbc4, treePL 1.0, Treeshrink 1.3.9. The summary of the alignments was produced with AMAS 1.0. The diversification analyses were performed with BAMM 2.5.0 and RevBayes 1.2.1.<br><br>The complete code used and developed to perform analyses is available in Zenodo (https://doi.org/10.5281/zenodo.10778206).

For manuscripts utilizing custom algorithms or software that are central to the research but not yet described in published literature, software must be made available to editors and reviewers. We strongly encourage code deposition in a community repository (e.g. GitHub). See the Nature Portfolio guidelines for submitting code & software for further information.

Data

Policy information about availability of data

All manuscripts must include a data availability statement. This statement should provide the following information, where applicable:

- Accession codes, unique identifiers, or web links for publicly available datasets
- A description of any restrictions on data availability
- For clinical datasets or third party data, please ensure that the statement adheres to our policy

All raw DNA sequence data generated for this study are deposited in the European Nucleotide Archive under the following bioprojects PRJNA478314, PRJEB35285, PRJEB49212 and PRJNA678873. All analysed data and metadata are available in Zenodo (https://doi.org/10.5281/zenodo.10778206). The resulting trees and metadata are also available in GBIF (https://doi.org/10.15468/4njn8b) and Open Tree of Life (https://tree.opentreeoflife.org/curator/study/view/ot_2304). The names used in this work match the World Checklist of Vascular Plants (https://doi.org/10.34885/jdh2-dr22).

Research involving human participants, their data, or biological material

Policy information about studies with human participants or human data. See also policy information about sex, gender (identity/presentation), and sexual orientation and race, ethnicity and racism.

Reporting on sex and gender	Not applicable
Reporting on race, ethnicity, or other socially relevant groupings	Not applicable
Population characteristics	Not applicable
Recruitment	Not applicable
Ethics oversight	Not applicable

Note that full information on the approval of the study protocol must also be provided in the manuscript.

Field-specific reporting

Please select the one below that is the best fit for your research. If you are not sure, read the appropriate sections before making your selection.

☐ Life sciences ☐ Behavioural & social sciences ☒ Ecological, evolutionary & environmental sciences

For a reference copy of the document with all sections, see nature.com/documents/nr-reporting-summary-flat.pdf

Ecological, evolutionary & environmental sciences study design

All studies must disclose on these points even when the disclosure is negative.

Study description	We sampled nearly 8,000 genera and 353 genes to infer a phylogenetic tree for angiosperms, dated and calibrated it with a dataset of 200 fossils, and used this evolutionary time frame to study the diversification of the group.
Research sample	We aimed to produce a dataset with at least one species per genus for >50% of the ca. 13,600 currently angiosperms genera. For genera with multiple species available, we retained up to three species, selecting primarily by phylogenetic representation followed by amount of data (number of genes and total length of recovery). Twelve species of gymnosperms were used as outgroups, one from each family. The final dataset contains 9,506 species, 7,923 genera, 416 families and 64 orders of angiosperms. It comprises 7,561 samples with data produced by this project and 1,963 samples mined from public repositories.
Sampling strategy	We sourced samples from herbarium, living, DNA bank and and tissue bank collections.
Data collection	We produced target sequence capture data using the Angiosperms353 probe kit. Total DNA was extracted using CTAB protocol and quantified by fluorometry. Average fragment size was assessed with 1% agarose gel. DNA extracts were diluted to 4 ng/ul. For extracts with high molecular weight, total DNA was fragmented using Covaris M220 Focused ultrasonicator. Genomic DNA libraries were prepared using NEBNext Ultra II DNA Library Prep Kit, following manufacturer's protocol at half volume, and with dual indexing. Libraries were normalised to 10 nM and pooled in equimolar amounts according to the average fragment size and taxonomic groups. Pools included, on average, 20-24 libraries. The pools were hybridised with Angiosperms353 probe kit. Hybridised pools were normalised to 7nM and combined for sequencing. Sequencing was carried out in Illumina platforms MiSeq and HiSeq.
Timing and spatial scale	Data production started in May 2016 and ended in December 2021.
Data exclusions	We excluded samples that failed both phylogenetic and barcode validation, as described in Baker (2022). Syst. Biol. 71: 301-319. We

Data exclusions	also excluded samples if more than three accessions were available for the same genus.
Reproducibility	All raw data and intermediate files are provided, making every step reproducible. All accessions used are listed in the Supplementary Table 1.
Randomization	Not applicable - randomization was not required.
Blinding	Not applicable - this work does not involve trials or controlled experiments.

Did the study involve field work? ☐ Yes ☒ No

Reporting for specific materials, systems and methods

We require information from authors about some types of materials, experimental systems and methods used in many studies. Here, indicate whether each material, system or method listed is relevant to your study. If you are not sure if a list item applies to your research, read the appropriate section before selecting a response.

Materials & experimental systems

n/a	Involved in the study	
☒	☐	Antibodies
☒	☐	Eukaryotic cell lines
☒	☐	Palaeontology and archaeology
☒	☐	Animals and other organisms
☒	☐	Clinical data
☒	☐	Dual use research of concern
☐	☒	Plants

Methods

n/a	Involved in the study	
☒	☐	ChIP-seq
☒	☐	Flow cytometry
☒	☐	MRI-based neuroimaging
