# Supplementary material for: Phylogenomics and the rise of the angiosperms
Source: Nature. 2024 Apr 24;629(8013):843–50. doi: 10.1038/s41586-024-07324-0 (PMC11111409; doi:10.1038/s41586-024-07324-0)
Supplement: Supplementary file 4 — Supplementary material [file 41586_2024_7324_MOESM4_ESM.zip › Supplementary_material/Supplementary_Fig_24.pdf]

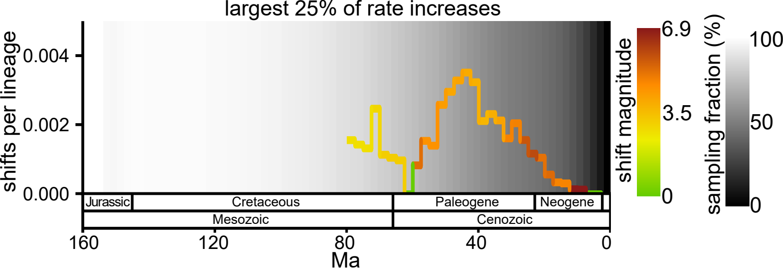
largest 25% of rate increases
shifts per lineage
0.004
0.002
0.000
Jurassic
Cretaceous
Paleogene
Neogene
Mesozoic
Cenozoic
160
120
80
40
0
Ma
shift magnitude
6.9
3.5
0
sampling fraction (%)
100
50
0
