# Supplementary figures and images for: Phylogenomics and the rise of the angiosperms
Source: Nature. 2024 Apr 24;629(8013):843–50. doi: 10.1038/s41586-024-07324-0 (PMC11111409; doi:10.1038/s41586-024-07324-0)

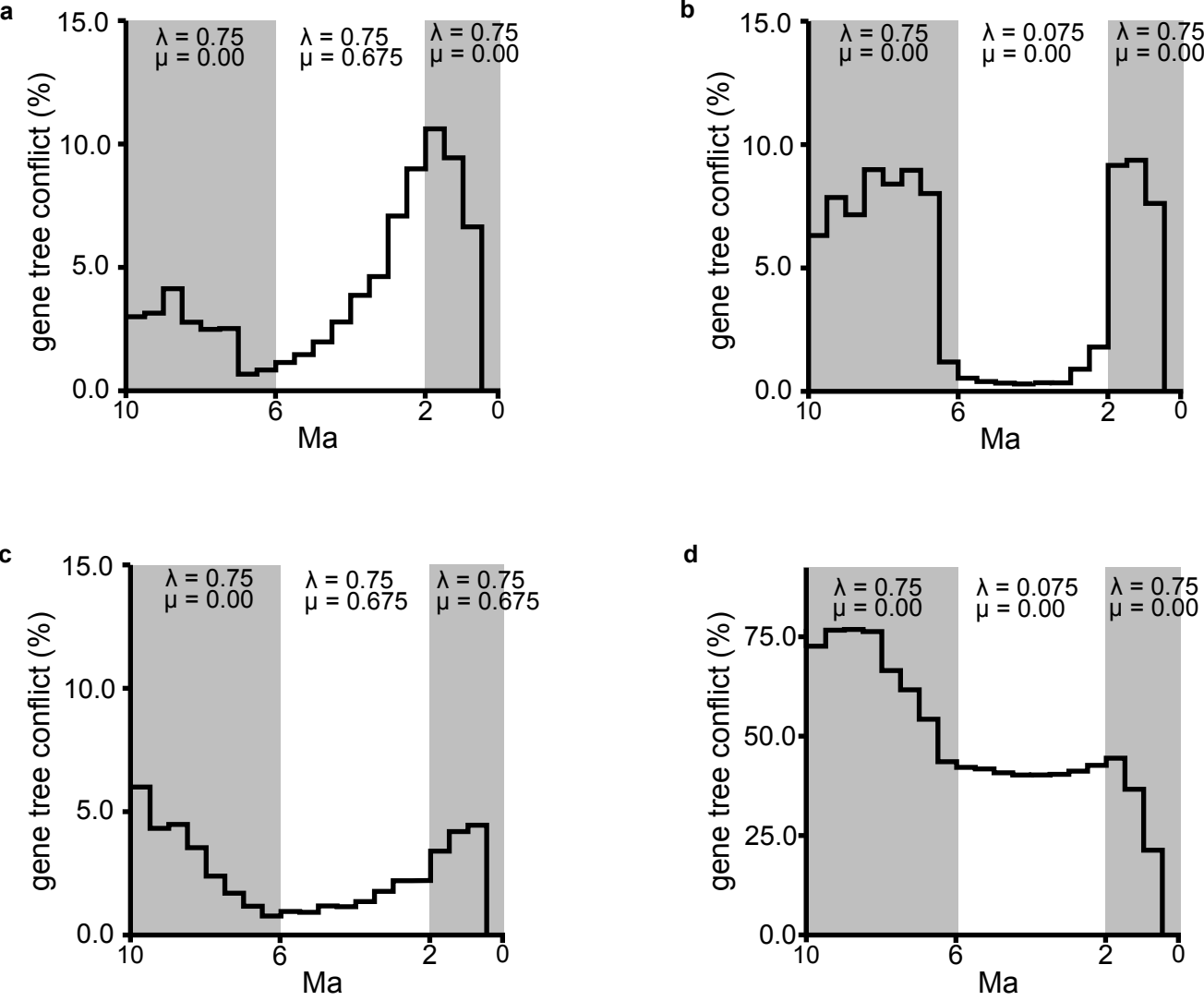

Supplement: Supplementary file 4 — Supplementary material [file 41586_2024_7324_MOESM4_ESM.zip › Supplementary_material/Supplementary_Fig_7.pdf]

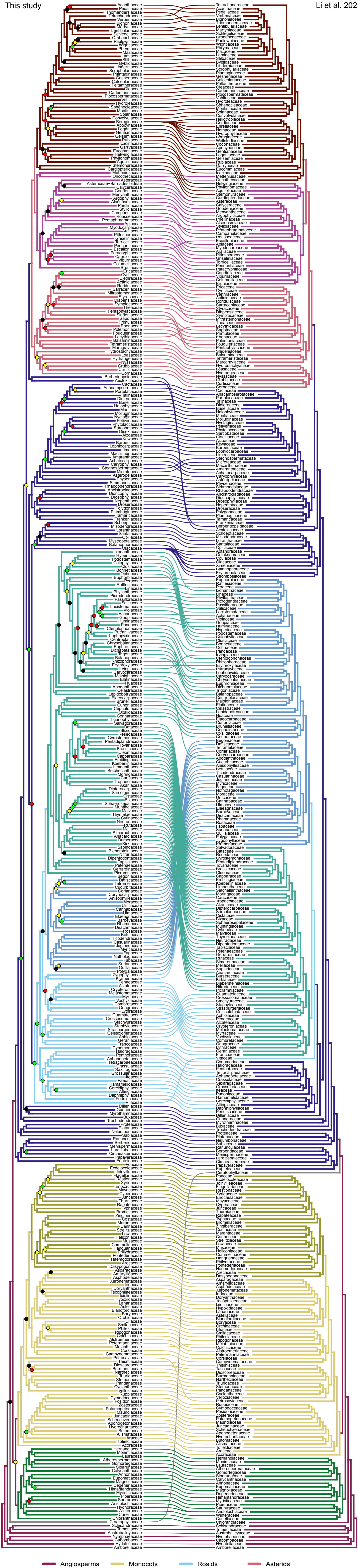

Supplement: Supplementary file 4 — Supplementary material [file 41586_2024_7324_MOESM4_ESM.zip › Supplementary_material/Supplementary_Fig_4.pdf]

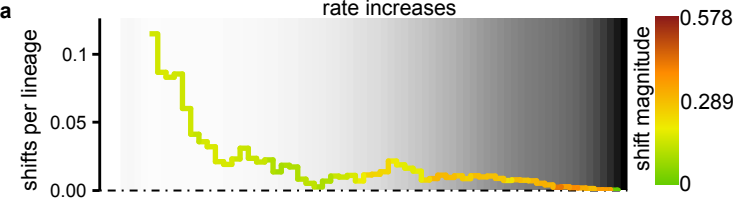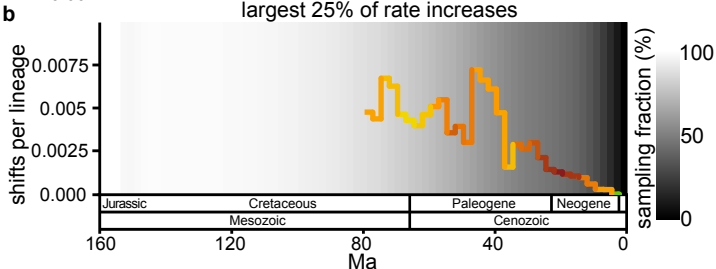

Supplement: Supplementary file 4 — Supplementary material [file 41586_2024_7324_MOESM4_ESM.zip › Supplementary_material/Supplementary_Fig_15.pdf]

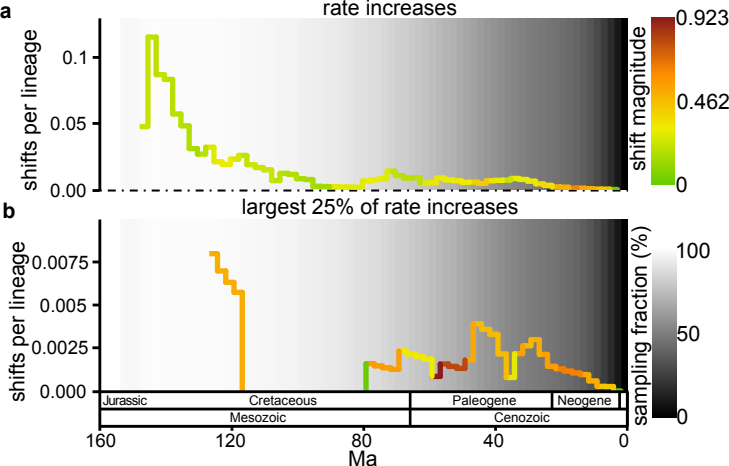

Supplement: Supplementary file 4 — Supplementary material [file 41586_2024_7324_MOESM4_ESM.zip › Supplementary_material/Supplementary_Fig_16.pdf]

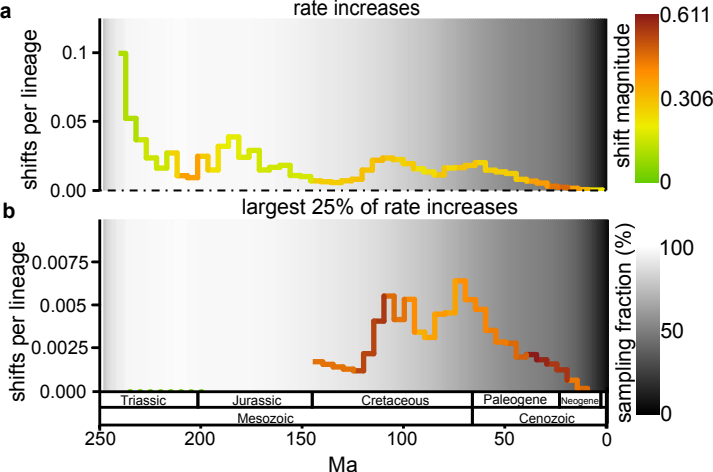

Supplement: Supplementary file 4 — Supplementary material [file 41586_2024_7324_MOESM4_ESM.zip › Supplementary_material/Supplementary_Fig_17.pdf]

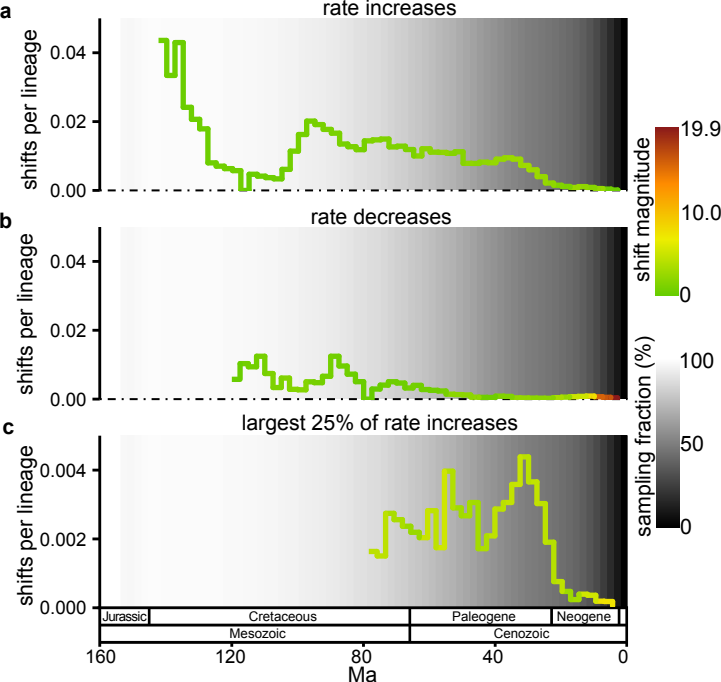

Supplement: Supplementary file 4 — Supplementary material [file 41586_2024_7324_MOESM4_ESM.zip › Supplementary_material/Supplementary_Fig_14.pdf]

Normalised Robinson-Foulds distance

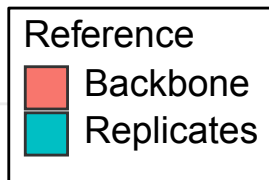

Backbone

Reference

Replicates

0.07

0.06

0.05

0.04

0.03

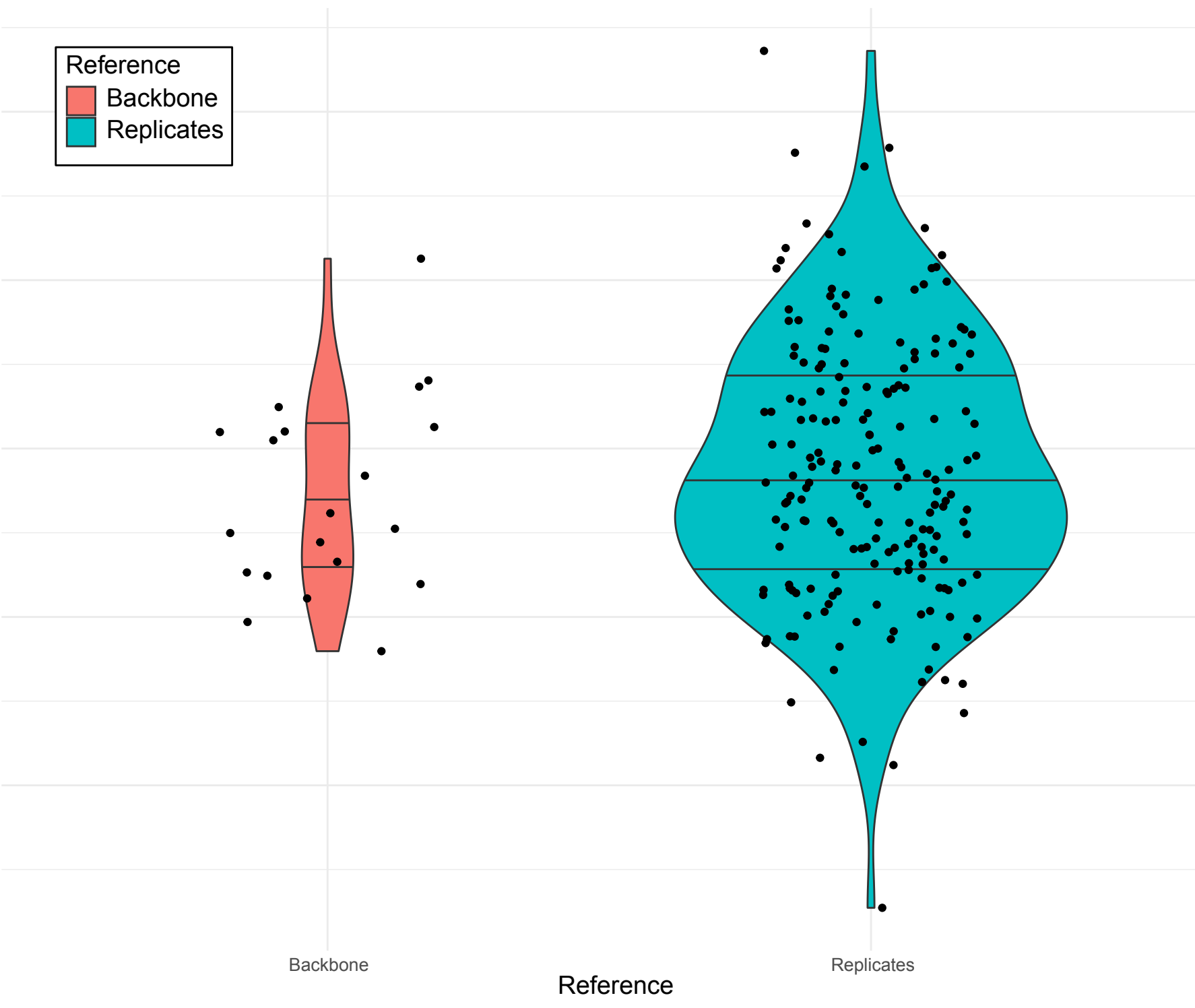

Supplement: Supplementary file 4 — Supplementary material [file 41586_2024_7324_MOESM4_ESM.zip › Supplementary_material/Supplementary_Fig_10.pdf]

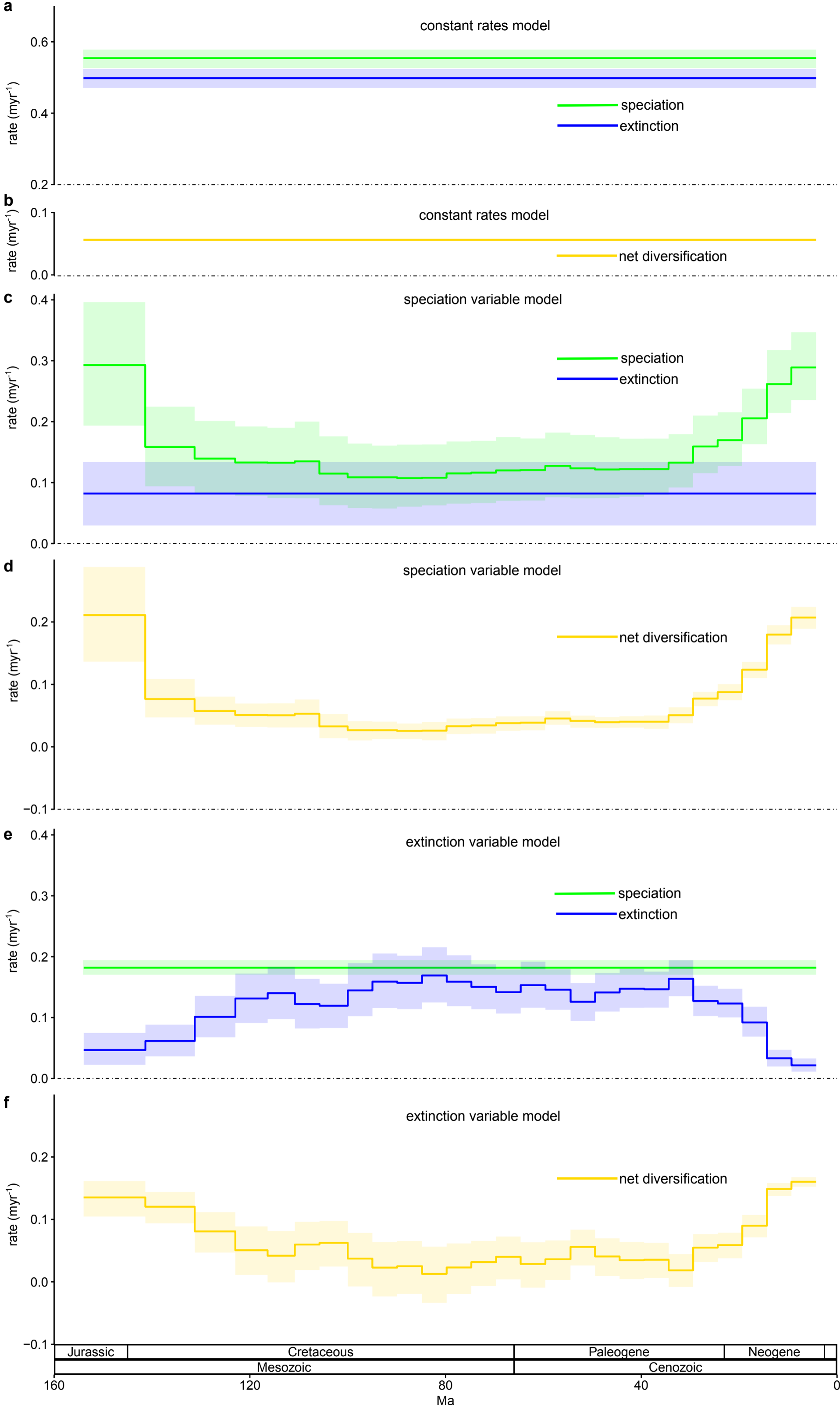

Supplement: Supplementary file 4 — Supplementary material [file 41586_2024_7324_MOESM4_ESM.zip › Supplementary_material/Supplementary_Fig_13.pdf]

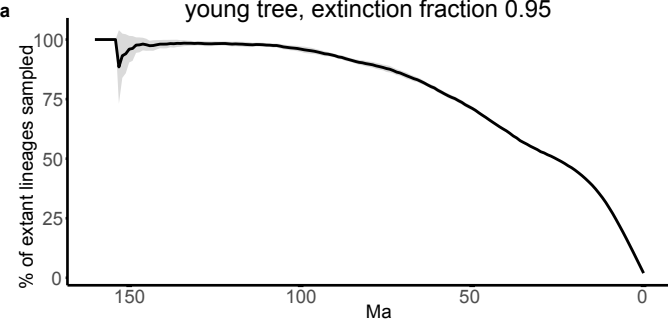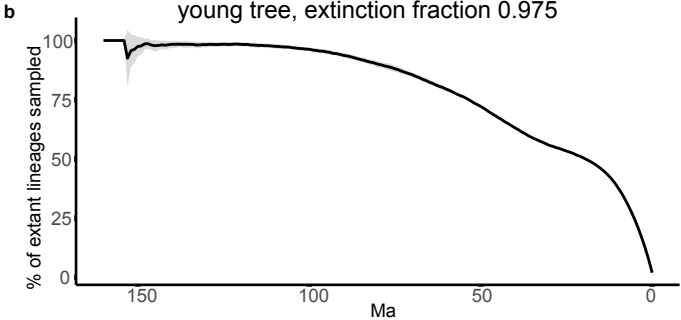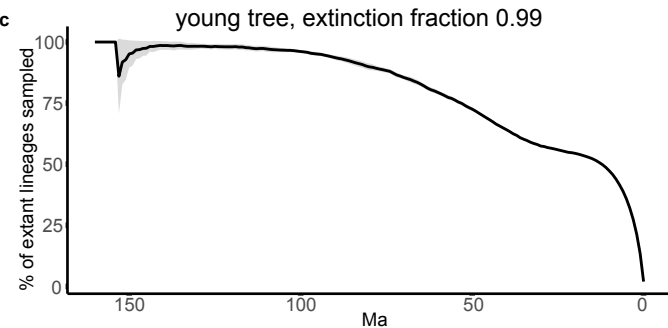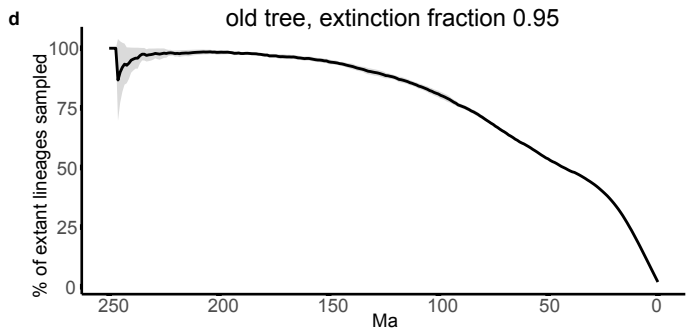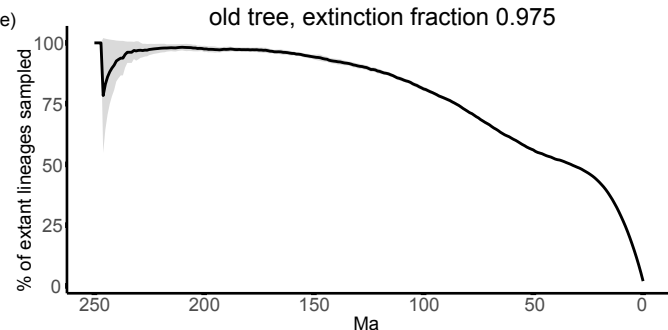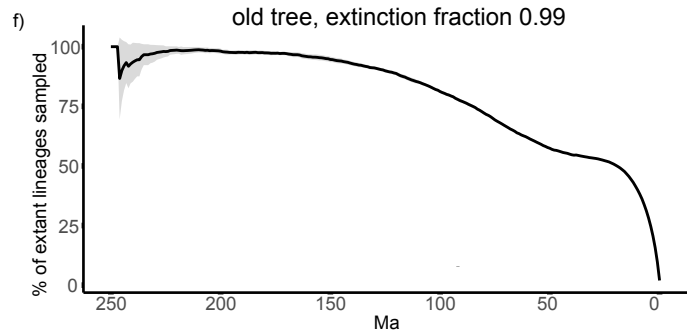

Supplement: Supplementary file 4 — Supplementary material [file 41586_2024_7324_MOESM4_ESM.zip › Supplementary_material/Supplementary_Fig_6.pdf]

## Backbone analysis

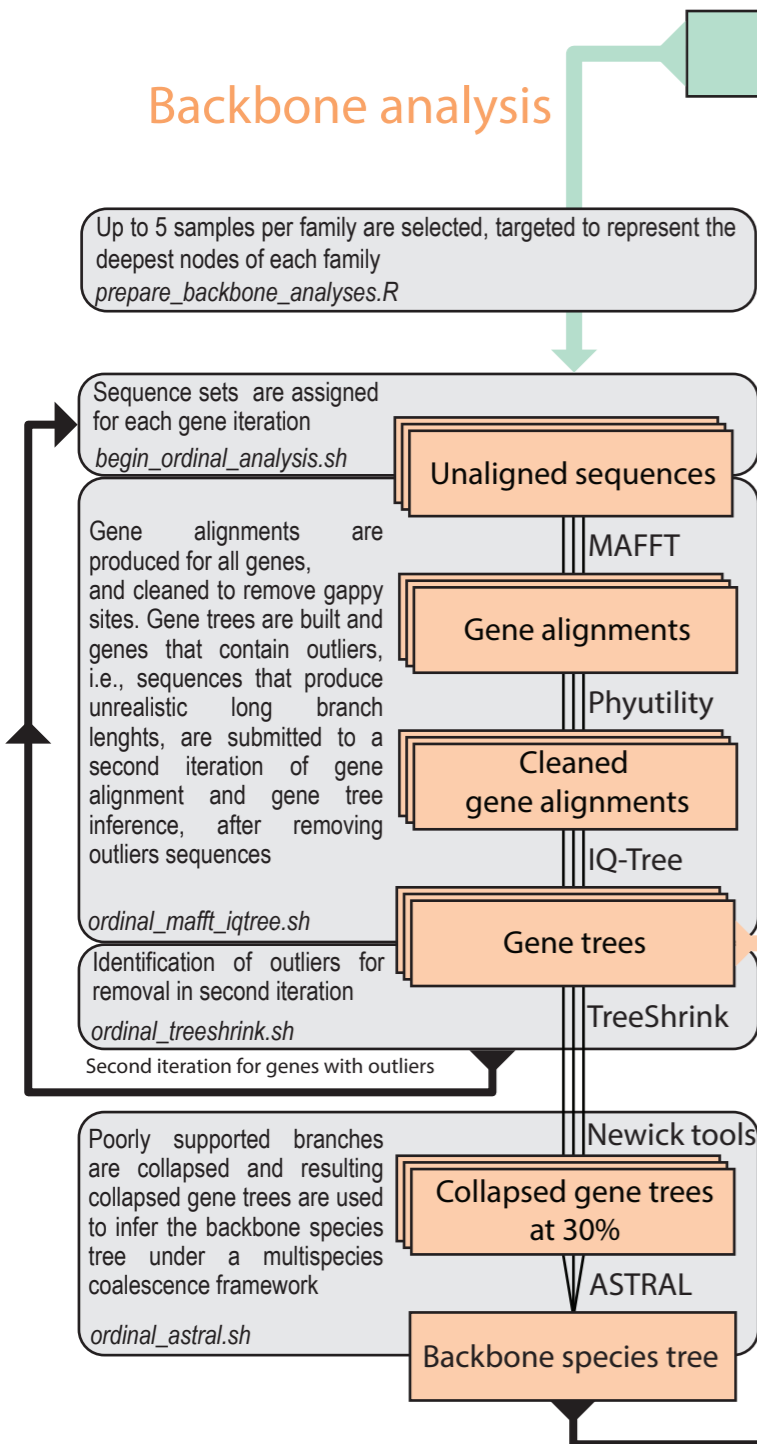

## Order-level sub-alignments

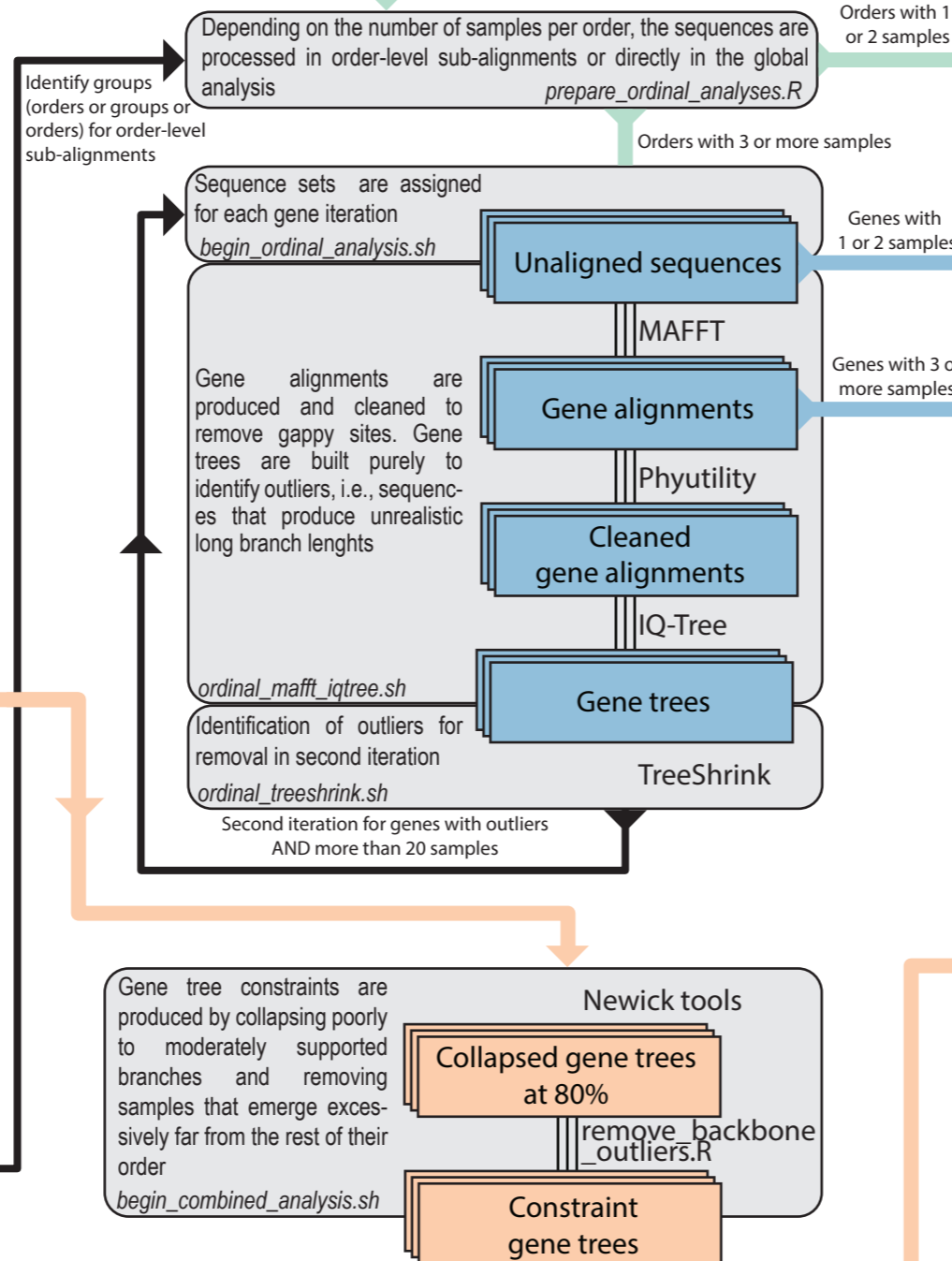

## Global analysis

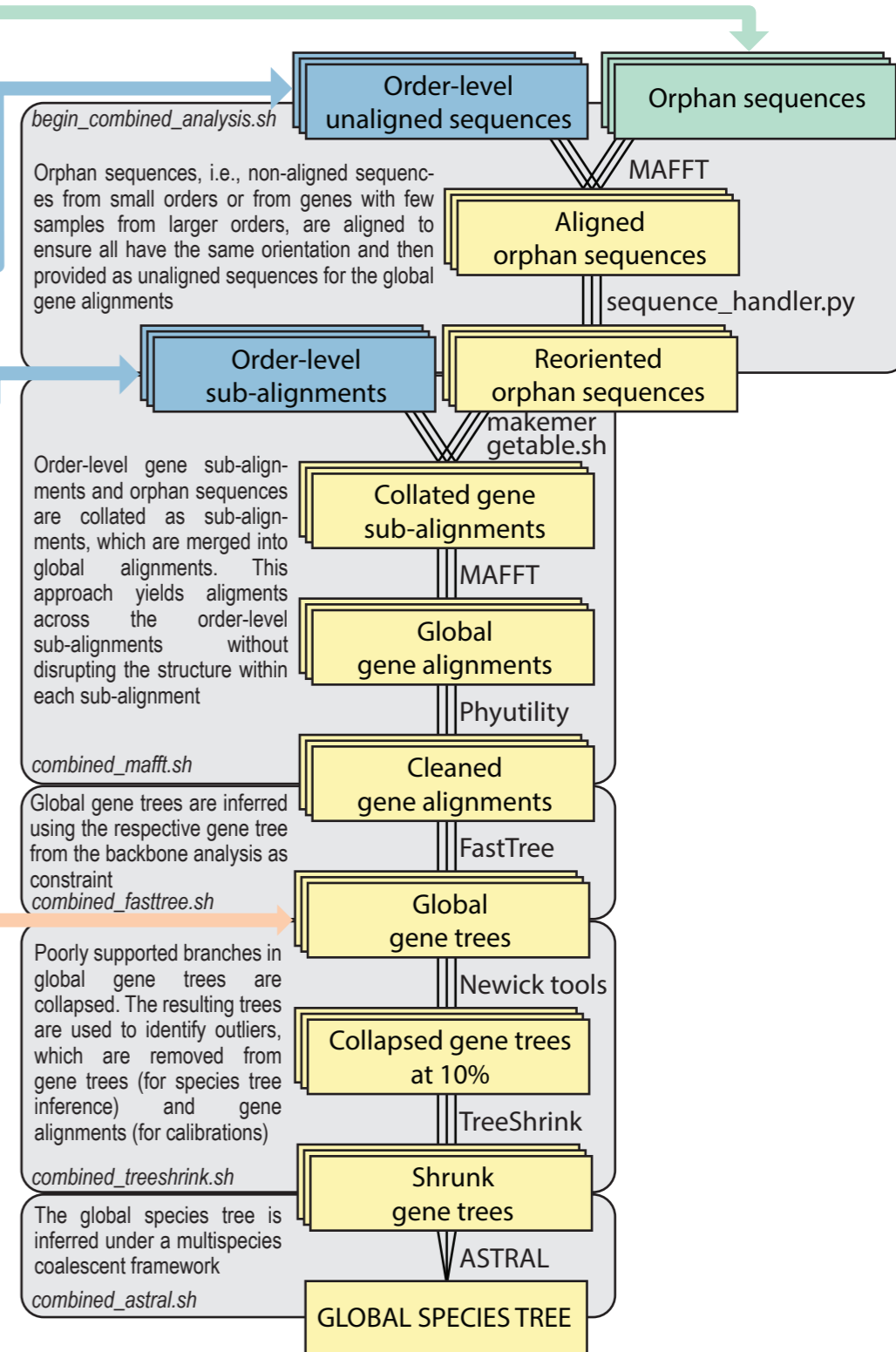

Supplement: Supplementary file 4 — Supplementary material [file 41586_2024_7324_MOESM4_ESM.zip › Supplementary_material/Supplementary_Fig_1.pdf]

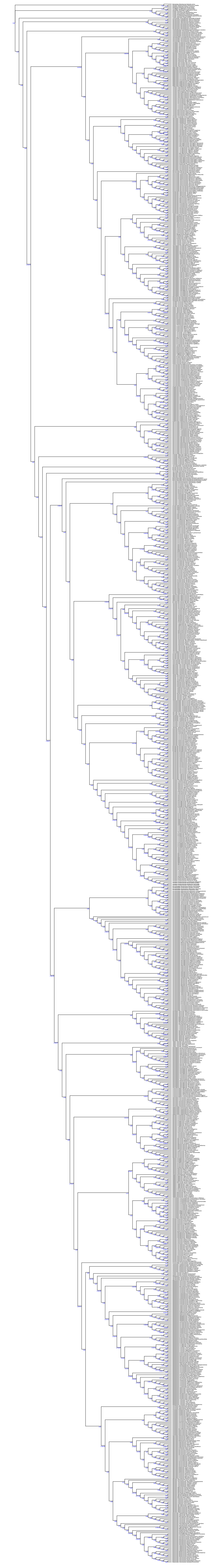

Supplement: Supplementary file 4 — Supplementary material [file 41586_2024_7324_MOESM4_ESM.zip › Supplementary_material/Supplementary_Fig_2.pdf]

Average  
occupancy

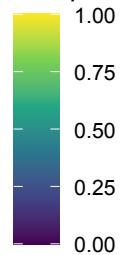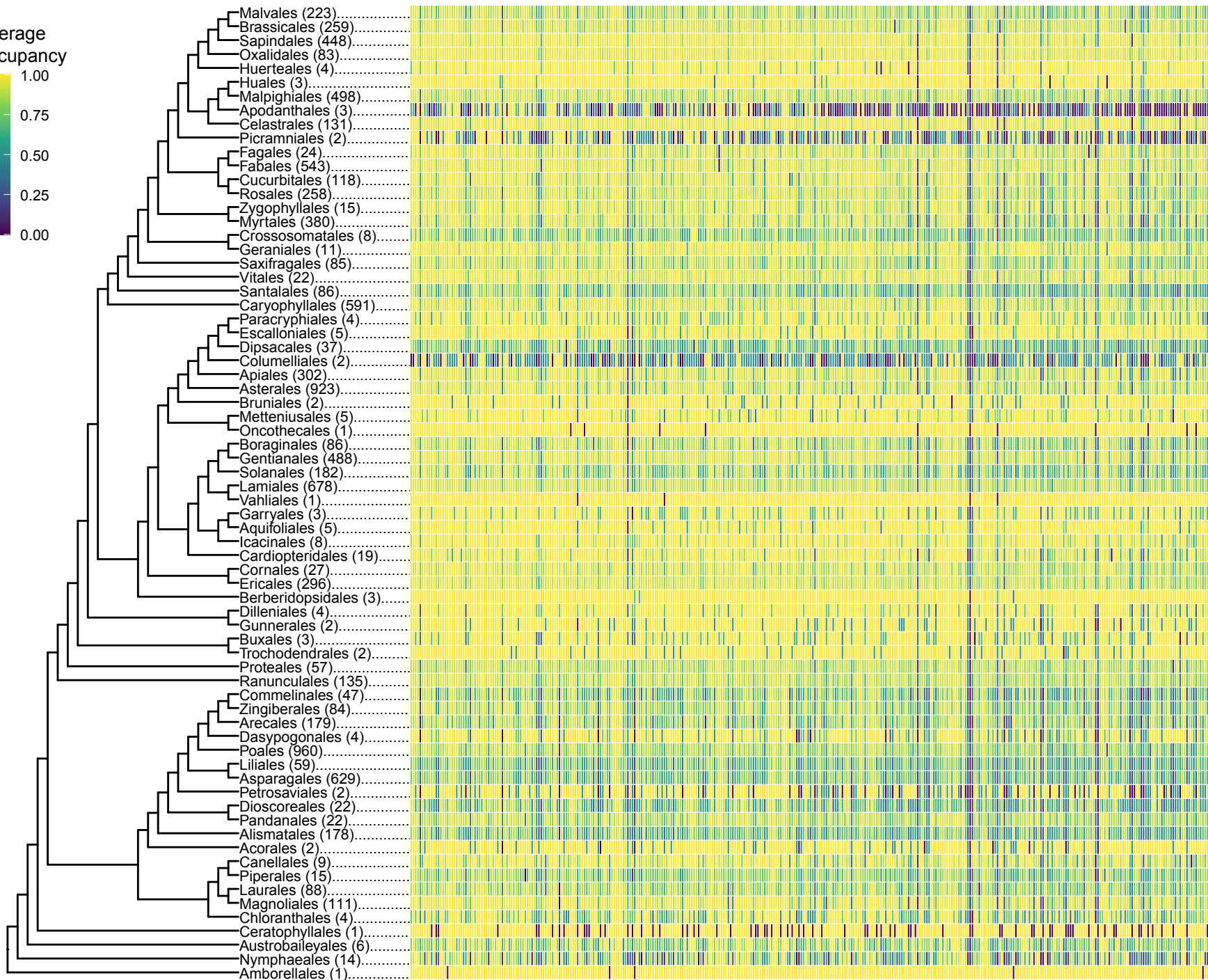

Supplement: Supplementary file 4 — Supplementary material [file 41586_2024_7324_MOESM4_ESM.zip › Supplementary_material/Supplementary_Fig_11.pdf]

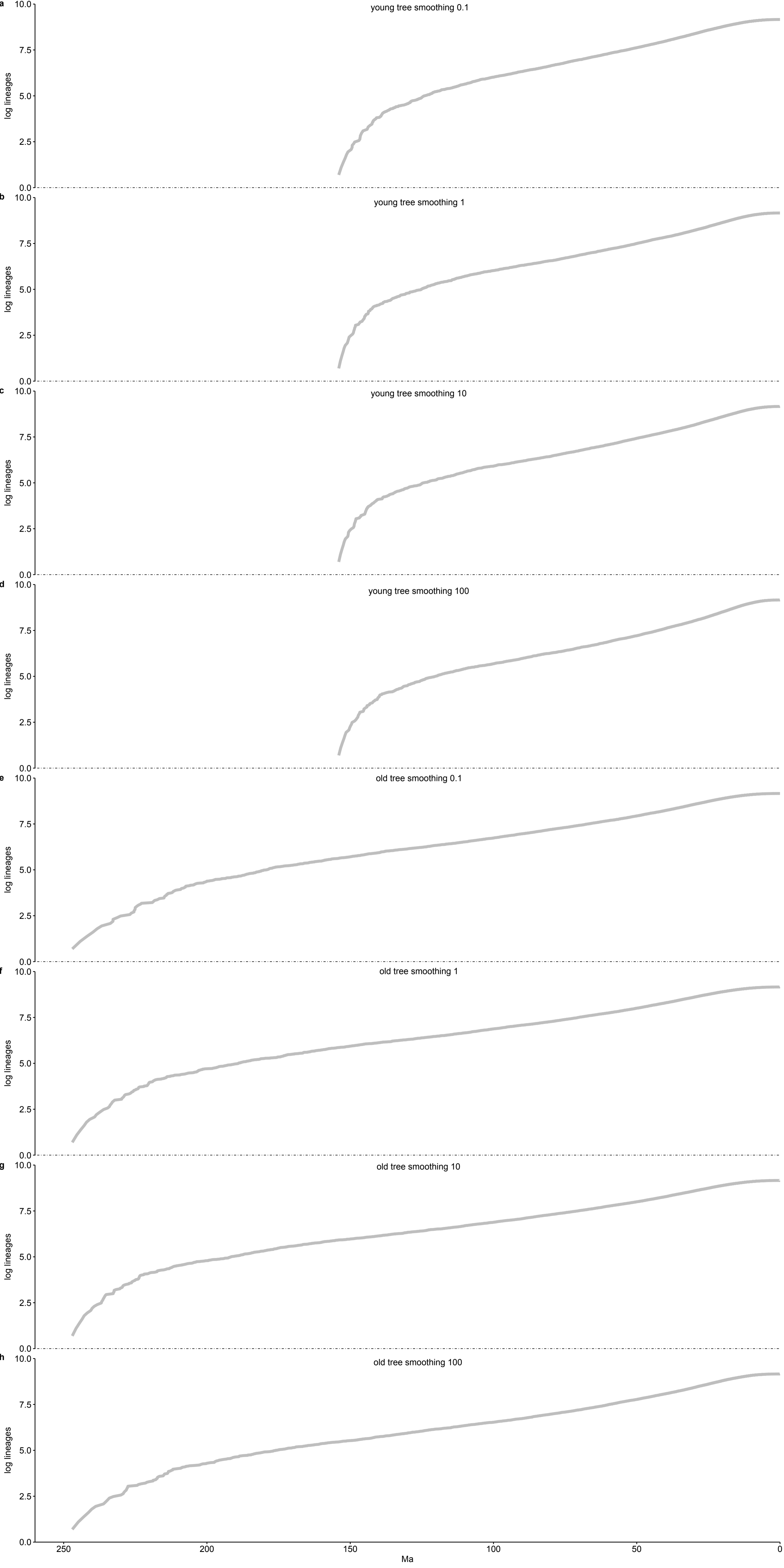

Supplement: Supplementary file 4 — Supplementary material [file 41586_2024_7324_MOESM4_ESM.zip › Supplementary_material/Supplementary_Fig_5.pdf]

Average  
recovery

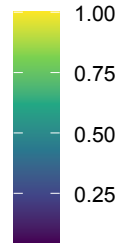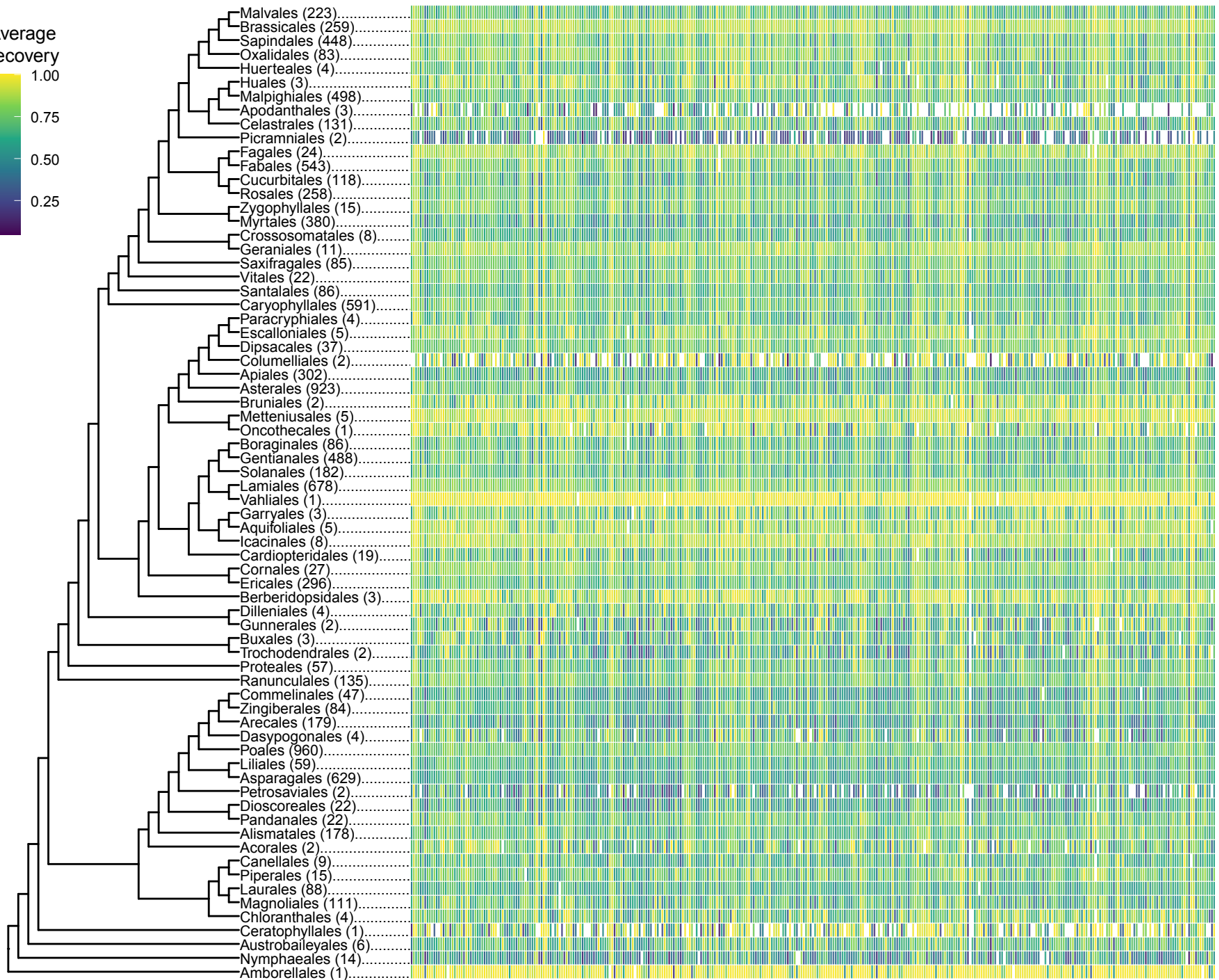

Supplement: Supplementary file 4 — Supplementary material [file 41586_2024_7324_MOESM4_ESM.zip › Supplementary_material/Supplementary_Fig_9.pdf]
