# Supplementary material for: Phylogenomics and the rise of the angiosperms
Source: Nature. 2024 Apr 24;629(8013):843–50. doi: 10.1038/s41586-024-07324-0 (PMC11111409; doi:10.1038/s41586-024-07324-0)

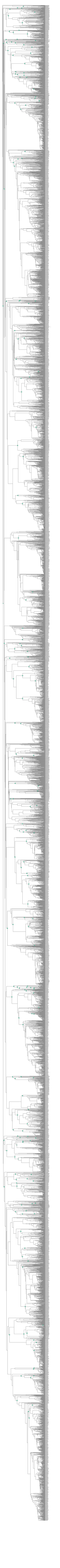

Supplement: Supplementary file 4 — Supplementary material [file Supplementary_Fig_12.pdf › Supplementary_material/Supplementary_Fig_12.pdf]
