# Supplementary material for: Phylogenomics and the rise of the angiosperms
Source: Nature. 2024 Apr 24;629(8013):843–50. doi: 10.1038/s41586-024-07324-0 (PMC11111409; doi:10.1038/s41586-024-07324-0)
Supplement: Supplementary file 4 — Supplementary material [file Supplementary_Fig_19.pdf › Supplementary_material/Supplementary_Fig_19.pdf]

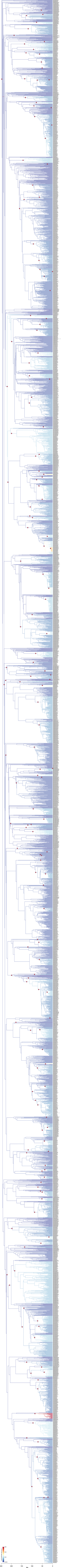
net diversification rate (myr⁻¹)
0.88
0.58
0.29
0.0033
250
200
150
100
50
0
Ma
